# Extracellular Vesicle Protein Cargo Reveals Potential Long-Term Biological Impact of Long COVID

**DOI:** 10.3390/cells15211969

**Published:** 2026-10-29

**Authors:** Kyosuke Oda, Arnel Belmonte, David Boulifard, Silvia Jakubski, Lindsey White, Charmagne Beckett, Andrew Letizia, Chad Porter, Eileen Villasante, Yuliya S. Johnson, Wathsala Wijayalath

**Affiliations:** 1 Henry M Jackson Foundation for Advancement of Military Medicine, Inc., Bethesda, MD 20817, USAarnel.d.belmonte.ctr@health.mil (A.B.); david.a.boulifard.ctr@health.mil (D.B.); silvia.j.jakubski.ctr@health.mil (S.J.); lindsey.j.white3.ctr@health.mil (L.W.); 2 Agile Vaccines and Therapeutics Department, Infectious Diseases Directorate, Navy Medicine Research and Development Command, Silver Spring, MD 20910, USA; eileen.villasante@gmail.com (E.V.); yuliya.s.johnson.mil@health.mil (Y.S.J.); 3 Translational and Clinical Research Department, Infectious Diseases Directorate, Navy Medicine Research and Development Command, Silver Spring, MD 20910, USA; charmagne.beckett@pharmaron-us.com (C.B.); letiziamd@yahoo.com (A.L.); chad.k.porter2.civ@health.mil (C.P.); 4 Division of Infectious Diseases, Department of Medicine, Jacobs School of Medicine and Biomedical Sciences, University at Buffalo, Buffalo, NY 14203, USA; 5 U.S. Naval Medical Research Unit SOUTH, U.S. Embassy Lima, Centro Medico Naval, Av. Venezuela cuadra 36 s/n, Callao 15088, Peru

**Keywords:** long COVID, Post-Acute Sequelae of SARS-CoV-2 (PASC), extracellular vesicles (EVs), peripheral blood mononuclear cells (PBMCs), proteomics, oxidative stress, immunosuppression, platelet dysfunction, biomarkers, cellular senescence

## Abstract

**Highlights:**

**What are the main findings?**

- PBMC-EV proteome signatures represent long-term impact of distinct Post-Acute Sequelae of SARS-CoV-2 (PASC) or long COVID symptoms.

**What are the implications of the main findings?**

- PBMC-EV metrics can serve as diagnostic, therapeutic and phenotypic biomarkers.
- PBMC-EVs can be used to investigate biological pathways underlying complex diseases such as long COVID.

**Abstract:**

Extracellular vesicles (EVs) carry status quo messages across the body that can help decode the long-term impact of Post-Acute Sequelae of SARS-CoV-2 infection (PASC). Here, we analyzed paired peripheral blood mononuclear cell (PBMC)-EVs (pre- and 6–8 months post-infection) collected from a subset of young United States (US) Marines with and without a history of PASC symptoms lasting >30 days post-infection, who participated in the COVID-19 Health Action and Response for Marines (CHARM) studies. Integrated nanospray LC-MS/MS, STRING and Reactome analysis of PBMC-EVs identified distinct proteomic signatures and biological pathways associated with different PASC symptoms. An individual who reported PASC headache and drowsiness (*n* = 1) had elevated histones, prelamin A/C, lysozyme C and S100-A9, among other proteins enriched with cellular stress pathways, including DNA damage, oxidative/metabolic/cytoskeletal stress, senescence, neutrophil degranulation and neurodegenerative-like diseases. Conversely, some participants who reported pulmonary PASC (*n* = 5, pooled) had elevated histones but decreased fibrinogen, integrin β-3 and S100-A9, mapped to senescence, platelet dysfunction, and immune exhaustion pathways. Non-PASC individuals (*n* = 9) exhibited active inflammation resolution, without returning to the pre-infection homeostasis even after 6–8 months post-infection. These distinct PBMC-EV proteomic signatures suggest potential long-term impact of long COVID with implications for the public health and mission readiness of US service members.

## 1. Introduction

The Coronavirus Disease-19 (COVID-19) pandemic, caused by the severe acute respiratory syndrome coronavirus 2 (SARS-CoV-2), resulted in a global health crisis. While many individuals fully recover from the acute infection, a significant proportion experiences persistent and debilitating symptoms for weeks, months, or even years after the initial infection. This lingering condition is now termed long COVID, or Post-Acute Sequelae of SARS-CoV-2 infection (PASC) [1]. We will use the terms long COVID and PASC interchangeably herein. PASC is characterized by a wide range of symptoms, including fatigue, dyspnea, cognitive dysfunction (“brain fog”), chest pain, headache, pulmonary complications and cardiovascular abnormalities [1]. Despite the growing awareness, there is currently no reliable biological framework to distinguish the complex underlying pathophysiological changes imprinted by PASC [1,2]. Due to the heterogeneity of PASC symptoms, existing approaches relying on imaging, localized, or compartmentalized analysis often fail to generate a scalable or mechanistically actionable framework for diagnosis or therapeutic interventions [3,4,5].

The long-term impact of PASC on quality of life in young adults (ages 18–35) has been significantly overlooked. In fact, one fourth of the healthy young United Sates (US) Marines who had asymptomatic or mild COVID-19 infection reported physical, cognitive, or psychiatric sequelae [6]. These data suggest that a considerable proportion of young adults suffer from PASC with decreased performance [6], a major concern for readiness and force health protection. This demographic, often characterized by active lifestyles and a lack of pre-existing chronic conditions, faces unique challenges in recognizing and reporting PASC symptoms. Difficulties in articulating these often subtle and evolving PASC symptoms, coupled with a general underestimation of the long-term consequences of even mild or asymptomatic COVID-19 infections, is clinically challenging. This situation is further compounded by incomplete vaccination status, potentially exacerbating the risk and severity of PASC. Existing clinical assessments rely heavily on subjective reporting, with limited molecular insight into disease heterogeneity or progression. Without clarity on actionable molecular signatures, clinicians and researchers are constrained in their ability to diagnose, stratify, or to create evidence-based treatment protocols and more personalized therapeutic approaches.

Emerging evidence indicates that PASC symptoms are driven by a complex, interactive network of molecular disruptions rather than a single, well-defined biological mechanism [1,2,3,4,5], one that is unlikely to be captured by compartmentalized analytical tools. Extracellular vesicles (EVs) offer a powerful, non-invasive platform to address this challenge. EVs are biological messengers found in, and secreted by, almost all cell types [7,8]. Based on their biogenesis pathways, EVs are classified into three major types: exosomes (30 nm–200 nm, derived from the endosomal trafficking pathway), micro-vesicles (MVs) (100–1000 nm, derived by outward budding of the plasma membrane) and apoptotic bodies (>1000 nm, released by cells undergoing programmed death) [7,8]. These heterogeneous non-replicating vesicles encapsulated by a lipid bilayer carry a diverse biological cargo rich in proteins, lipids, metabolites and nucleic acids (DNA, mRNA, microRNAs, and other non-coding RNAs), reflecting the physiological state of their donor cell [7,8]. Crucially, EVs are not confined to a single tissue or compartment. They traverse biological barriers, sample from diverse donor cells, and deliver cargo-encoded molecular messages across local and distant organ systems. Thus, EVs form a dynamic communication network that reflects the integrated physiological state of the entire body. This makes them unparalleled tools for capturing whole-organism biological signatures, surpassing many traditional compartmentalized omics approaches that rely on single cells or tissue-specific snapshots. By extracting and analyzing EV cargo from accessible fluids or cells [e.g., plasma, urine, saliva, peripheral blood mononuclear cells (PBMCs)], researchers can map complex biological networks, monitor signaling pathway activity, and detect early disease-associated perturbations without the need for invasive biopsies. While the exact molecular mechanisms of EV recognition and cellular uptake are unknown, the current evidence supports EV internalization through receptor–ligand interactions, endocytosis or direct fusion with plasma membrane after attaching to the cell surface [7,8].

Prior investigations have focused on EVs as carriers of viral and host biomarkers in long COVID, employing targeted approaches such as flow cytometry, immunophenotyping, and protein quantification via enzyme-linked immunosorbent assay (ELISA) or Western blot, primarily in neuron- or astrocyte-derived EVs or plasma-isolated EVs [9,10,11,12,13,14,15]. While these studies have revealed persistent viral proteins, immune dysregulation, and organ-specific pathology, their scientific rigor is constrained by narrow analyte panels and pre-selected targets, limiting the discovery of novel mechanisms and systemic signatures. Above all, none of these studies have specifically analyzed EVs released from PBMCs (PBMC-EVs) to identify distinct proteins and biological pathways associated with long COVID. Hypothetically, PBMC-EVs offer enhanced cellular specificity, increased signal-to-noise ratio, and functional relevance, enabling precise mapping of molecular mechanisms. In contrast, commonly used plasma EVs represent a “snapshot” of virtually all tissues in the body facilitating biomarker discovery, while creating high heterogeneity that could obscure specific mechanisms. Also, plasma EVs co-purify with lipoproteins and soluble plasma proteins, which can interfere with downstream omics analysis and obscure biologically relevant signals. As such, PBMC-EVs could be more beneficial than plasma EVs for analyzing disease-associated biological pathways, especially those involving immune or inflammatory mechanisms.

In the present study, we used EVs enriched from human PBMCs to investigate the long-term health impact of PASC by characterizing the EV proteome via mass spectrometry. Paired PBMC samples (pre-infection and 6–8 months post-infection at the time of the PASC survey) used in this study had been previously collected from active-duty US Marines (aged 18–23) who were enrolled in the COVID-19 Health Action Response for Marines (CHARM 1.0 and 2.0) clinical studies [6,16]. At the 6–8 months PASC survey, participants were classified as having PASC if they reported symptoms that lasted for more than 30 days post-SARS-CoV-2 infection [6]. The present study captures the molecular signatures that potentially lingered up to 6–8 months after the initial SARS-CoV-2 infection. Despite the small cohort size, we found distinct proteomic signatures mapped to specific biological pathways in PBMC-EVs linked to participants with a history of different PASC symptoms. While it can be argued that these signatures may be a result of other interim infections or random biological fluctuations, the differential protein fold-changes and statistically enriched STRING and Reactome datasets suggest an altered biological footprint imprinted by PASC. We acknowledge that this is a *hypothesis-generating* rather than a mechanistic study. Particularly, the data related to the single participant who reported having PASC headache and drowsiness are considered as a case observation. Our findings establish a critical foundation for future investigations aimed at fully elucidating the biological mechanisms of PASC.

## 2. Materials and Methods

### 2.1. Samples

Use of de-identified human PBMC samples in this study was approved as non-human subjects research through a human subject research determination conducted by the Naval Medical Research Command (NMRC) Institutional Review Board in compliance with all applicable federal regulations governing the protection of human subjects.

Paired PBMC samples that had been collected before the initial SARS-CoV-2 infection (T0) [16] and at the time of PASC survey 6–8 months (181–259 days) post-SARS-CoV-2 infection (T1) [6] were used in the present study. This resulted in a total of 34 clinical samples analyzed across the 17 paired participants (T0 and T1 time points per individual). To ensure stringent quality control, one PASC individual (PASC8) was subsequently excluded from downstream proteomic and pathway analyses because their enriched EV fraction lacked detectable ExoCarta-annotated EV markers at the T1 time point. Consequently, protein interaction and biological pathway analyses were conducted on the remaining seven PASC individuals (PASC1–7) and non-PASC (*n* = 9) individuals. The sample size for this study was determined by the availability of paired (pre- and post-infection) specimens from the CHARM study.

**Figure 1:**
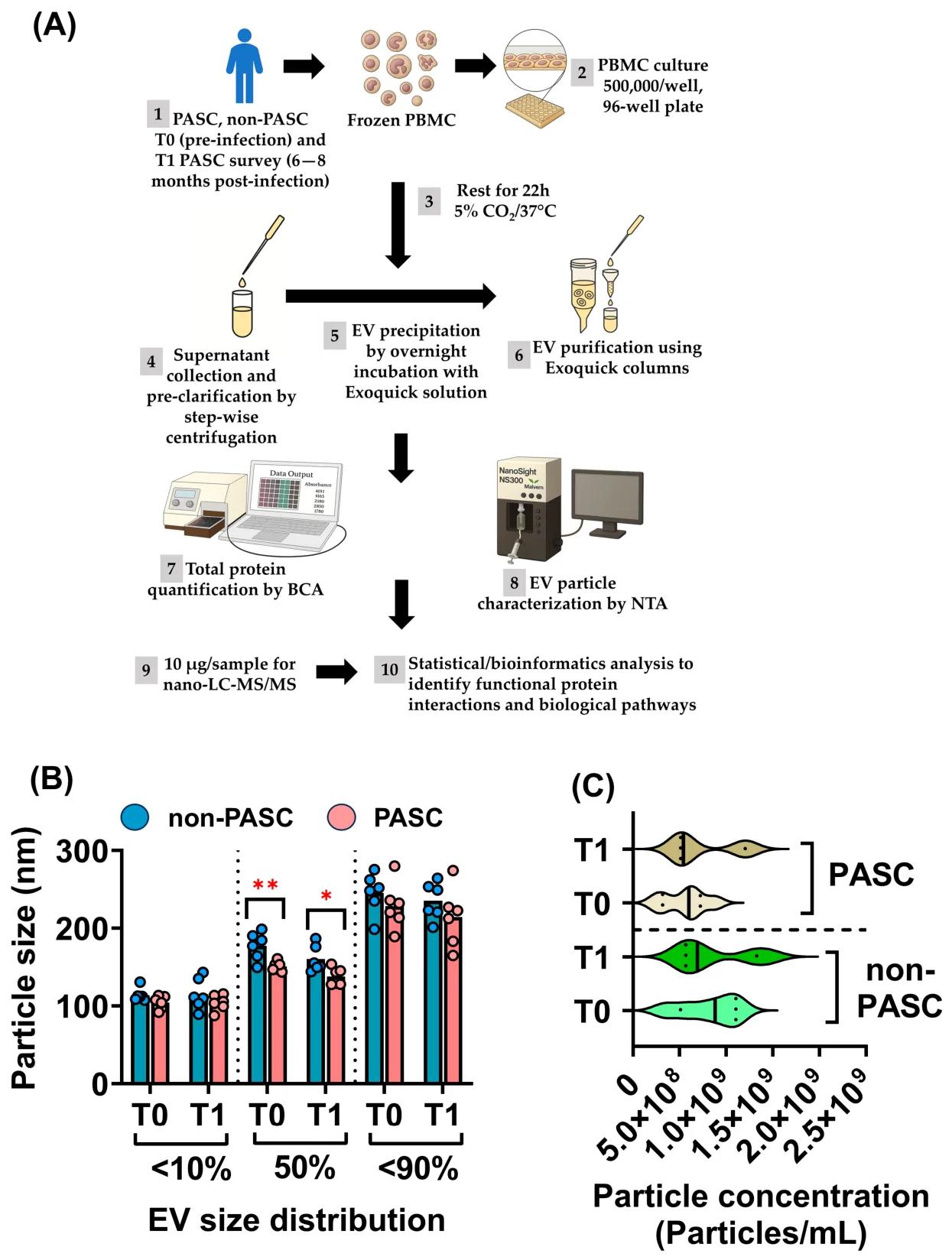
**PBMC-derived EV purification and characterization**. (**A**) Schematic representation of the PBMC-derived EV purification and nano-LC-MS/MS analysis workflow. Paired T0 (pre-infection) and T1 (6–8 months post-infection) with a history of PASC (*n* = 8) and non-PASC individuals (*n* = 9, without a history of long COVID symptoms) profiled by nano-LC-MS/MS after normalizing to the total protein quantity (10 µg/sample). The nano-LC-MS/MS protein hits were analyzed against human and SARS-CoV-2 proteomes. Significant protein hits were filtered by median combined FDR (≤0.05). (**B**) EV particle size distribution. <10% and <90% indicate the diameters (D) below which 10% (D10) and 90% (D90) of particles are observed and 50% (D50) represents the median diameter. Asterisks (*) denote significance at 0.05 alpha level, * *p* < 0.05, ** *p* < 0.01. Unpaired T test was used to compare between PASC and non-PASC after testing for normality. (**C**) Concentration of EV particles (particles/mL). The bold horizontal line represents the median value, while the upper and lower dashed lines indicate the 75th and 25th percentiles (interquartile range), respectively.

With the exception of one non-PASC individual (non-PASC3) who had completed their second dose of the SARS-CoV-2 vaccine at the time of the T1 survey, all other participants were unvaccinated at the time of the T1 PASC survey (Figure 1A, Table S1). Participant demographics are detailed in Supplementary Table S1.

To minimize selection bias, PASC and non-PASC participants were matched from the same parent perspective cohort (CHARM 1.0/2.0) [6,16] based on key demographic and clinical characteristics. As detailed in Table S1, the groups were balanced with respect to age, gender, smoking status, and the duration between pre- (T0) and post-infection (T1) sample collection. Furthermore, there were no systematic differences in baseline SARS-CoV-2 infection severity, with both symptomatic and asymptomatic individuals present in each group, ensuring that observed proteomic differences were not biased by the baseline.

### 2.2. PBMC Cultures and Ex Vivo EV Release

PBMCs were processed in different batches for EV isolation. Paired PBMC samples (T0 and T1) from the same individual were included in the same batch for consistency. Freeze-thawed PBMCs were cultured in complete culture medium composed of RPMI 1640 (Gibco, Grand Island, NY, USA, Cat. 22400105) supplemented with 10% exosome-depleted fetal bovine serum (Gibco, Cat. A2720801), 1X Pen-Strep-G (Gibco, Cat. 10378016), 1X Minimum Essential Medium Non-Essential Amino Acids (Gibco, Cat. 11140050), 40 mM L-Glutamine (Gibco, Cat. 25030081), 20 mM Sodium Pyruvate (Gibco, Cat. 11360070), and 0.05 mM 2-mercaptoethanol (Gibco, Cat. 21985023). Briefly, the frozen PBMC vials were placed in a 37 °C water bath for 2 min, immediately after removing the vials from the liquid nitrogen freezer. The partially thawed cell suspension from each PBMC vial was transferred to a 50 mL conical tube. Next, 10 mL of pre-warmed thawing medium (complete medium supplemented with 0.02% Benzonase (MilliporeSigma, Burlington, MA, USA, Cat. 71206-3)) was added dropwise to each cell suspension. Freeze-thawed PBMC suspensions were centrifuged at 2000 rpm for 5 min at room temperature (20–25 °C), followed by a wash with 10 mL of pre-warmed complete culture medium. PBMCs resuspended in complete culture medium were filtered through 70µM to remove any cell clumps. Cell counts were taken using the LUNA-FL dual fluorescence automated cell counter (Logos Biosystems, Annandale, VA, USA). PBMCs were immediately plated in 96-well, flat-bottom tissue culture plates (Corning, NY, USA, Cat. 3596) at a density of 500,000 cells/100 µL/well. Plates were incubated for at least 22 h at 5%CO_2_/37 °C to allow natural release (ex vivo) of EVs from PBMCs into the culture supernatant. Cell counts and viability are reported in Table S2.

### 2.3. EV Precipitation and Purification

PBMC-EVs were precipitated and purified using the ExoQuick-TC ULTRA EV isolation kit for tissue culture media per manufacturer’s instructions (System Biosciences, LLC., Palo Alto, CA, USA, Cat. EQULTRA-20TC-1). First, culture supernatant was collected (pooled if originated from the same PBMC sample) and sequentially centrifuged at 300 *g* for 10 min, 2000 *g* for 30 min and 10,000 *g* for 45 min at 4 °C. After each spin, supernatant was carefully collected to remove dead cells, cellular debris, apoptotic bodies and larger vesicles. After the final spin, each supernatant sample was transferred to a new low-protein binding tube. To precipitate EVs, ExoQuick-TC reagent (polymer-based) was added to each sample at 20% of the total volume of the supernatant and mixed vigorously by inverting the tubes. The supernatants were incubated for at least 12 h at 4 °C. Next, to purify precipitated EVs, the ExoQuick-TC/supernatant mixture was centrifuged at 3000 *g* for 10 min at 4 °C. Then, the supernatant was carefully removed without disturbing the pellets and each EV pellet (most of the time pellets were invisible) was sequentially resuspended in 200 µL of buffer B and 200 µL of buffer A (EV pellets from the same sample were pooled into Buffer B). EVs were purified and eluted in a mixture of buffer A and B (total eluted volume ~ 400–450 µL) per manufacturer’s instructions using the ExoQuick-TC resin columns. Briefly, purification columns were prepared by removing the storage buffer via centrifugation at 1000 *g* for 30 s, followed by two sequential washing steps with 500 μL of Buffer B at 1000 *g* for 30 s. After washing, the columns were loaded with 100 μL of Buffer B, and the EV-buffer mixture. The columns were incubated on a rotating shaker at room temperature for 5 min. Finally, the purified EVs were eluted directly into 2 mL low-protein binding microcentrifuge tubes by centrifuging the columns at 1000 *g* for 30 s. Purified EVs were aliquoted and stored at +4 °C (2–48 h for protein quantification and particle characterization) or were frozen at −80 °C for mass spectrometry analysis. All the EV precipitation/purification steps were conducted under aseptic conditions with pre-sterilized tubes/containers inside biosafety level II cabinets (except during centrifugations). Samples were handled with a double layer of gloves, sleeves and masks. All these steps were taken to prevent EV contamination from external sources such as skin, saliva, reagents, and equipment.

### 2.4. EV Total Protein Quantity Measurement

Total EV protein concentration was determined within 2 h after EV purification using the Pierce™ BCA Protein Assay Kit (Thermo Fisher Scientific, Waltham, MA, USA, Cat. 23227) per manufacturer’s instructions. Briefly, two independent aliquots (20 µL each) were taken from each purified EV sample for BCA assay after briefly sonicating the EV samples three times (10 s on/10 s off) in an ultrasonic water bath (Branson Ultrasonics CPX2800, Branson Ultrasonics Corporation, CT, USA). This ensured the homogeneity of aliquots and consistency of BCA readouts between the duplicates. Next, the purified EV aliquots were lysed with 20 µL of M-PER protein extraction reagent (Thermo Fisher Scientific, Cat. 78501) followed by 10 min sonication in “high” mode in the ultrasonic water bath. Lysed EV samples were briefly spun and mixed thoroughly before loading on to the 96-well assay plate (Immulon 2 HB, Thermo Fisher Scientific). Background control for the EV samples was prepared by mixing the elution buffer used for the EV purification with buffers A and B from the EV purification kit at the same ratio that was used for the EV purification. The elution buffer was then diluted 1:1 with the M-PER protein extraction reagent and loaded onto the 96-well plate. 1X phosphate-buffered saline (PBS) was also loaded onto the 96-well plate as a background control for the standards. The plate was sealed and mixed on the plate shaker for 30 s and incubated at 37 °C for 30 min. Optical density (OD) was measured at 562 nm using the Spectra max 354 plus plate reader. Background OD was subtracted from each sample using appropriate background controls (for samples and standards). A standard curve was generated by the GraphPad Prism, v10.1.2 software by fitting the OD measurements and respective concentrations of the serial dilutions of the BCA standards to sigmoidal 4-PL model (GraphPad Prism, v10.1.2). Total EV protein concentration was determined after interpolating the standard curve and applying the dilution factor. Based on the total EV protein concentration, 10 µg of total protein/sample was aliquoted for mass spectrometry analysis and immediately frozen at −80 °C. To ensure homogeneity, each stock EV sample was briefly sonicated three times (10 s on/10 s off) as described previously, before preparing aliquots for mass spectrometry. The remaining samples were stored at +4 °C for 24–48 h for nanoparticle tracking analysis.

### 2.5. Nanoparticle Tracking Analysis (NTA)

Nanoparticle tracking analysis (NTA) was used to determine the size distribution and concentration of the purified EV particles based on the principles of light scattering and Brownian motion. Samples were analyzed using a NanoSight NS300 instrument (Malvern Panalytical, Malvern, UK) per NanoSight NS300 user manual (NTA 3.2 Dev Build 3.2.16). First, the NS300 was calibrated using 200 nm and 400 nm latex size indicator transfer standard polystyrene beads (Thermo Fisher Scientific, Cat#NTA4089 and NTA4091). Prior to analysis, EV samples were briefly sonicated three times (10 s on/10 s off) as described previously. EV samples were then diluted in 1X PBS (2–4 times), loaded into 1 mL syringes and injected into the NanoSight NS300 using an automatic pump (Masterflex Single-Syringe Infusion Pump, Avantor; 115 VAC, Radnor, PA, USA, Cat. MFLX74900-00) at a rate of 0.4 mL/h to ensure a controlled sample flow. The viscosity setting was adjusted to match that of 1X PBS at 0.888cP. As the sample was being loaded into the instrument, screen gain, camera level, and the focus were adjusted based on the initial live image of the samples prior to capturing the video. Three 10 s captures were recorded per sample. Once the recordings were captured, size distribution and concentration matrices were generated by the software along with the graphs.

### 2.6. Nanospray Liquid Chromatography Coupled with Tandem Mass Spectrometry (Nano-LC/MS/MS) Analysis and Database Search

Nano-LC/MS/MS analysis and database searching were carried out by a commercial vendor (Poochon Scientific, Frederick, MD, USA). Frozen EV aliquots were shipped to the vendor on dry ice via overnight shipping. To minimize run-to-run variability and batch effects, samples were organized such that paired T0 and T1 samples from each individual were analyzed within the same instrument run, with PASC and non-PASC cohorts run in separate, sequential batches. EV samples (normalized to 10 µg total protein per sample) were first reduced with 10 mM dithiothreitol (DTT, Sigma-Aldrich, Inc., St. Louis, MO, USA) at 56 °C for 45 min, followed by alkylation with 25 mM iodoacetamide (Sigma-Aldrich, Inc.) for 30 min at room temperature in the dark. Alkylated proteins were then precipitated using 80% acetone. The protein mixtures were subsequently subjected to digestion with MS-grade trypsin (Pierce Trypsin Protease) using a trypsin-to-protein ratio of 1:50 (*w*/*w*) at 37 °C for 16 h. Following digestion, the peptide mixtures were concentrated utilizing a Speed Vacuum Concentrator (Thermo Savant ISS110, Thermo Fisher Scientific) and desalted using C18 ZipTips (MilliporeSigma, St. Louis, Missouri, Cat. ZTC18S960). Desalted peptides were reconstituted in 30 µL of 0.1% Optima LC/MS-grade formic acid (Fisher Scientific, Pittsburgh, PA, USA) prepared in nano-LC-MS/MS grade water (Sigma-Aldrich, Inc). A 10 µL aliquot of the reconstituted peptides was used for subsequent LC/MS/MS analysis.

Nano-LC/MS/MS analysis was performed using a Thermo Scientific Orbitrap Exploris 480 Mass Spectrometer coupled to a Thermo Vanquish Neo RSLCnano System. The peptide mixture from each sample was loaded onto a peptide trap cartridge (Thermo Scientific™ PepMap™ Neo Trap Cartridge, Catalog#174500) at a flow rate of 5 μL/min. Trapped peptides were then eluted onto a reversed-phase analytical column (Thermo Scientific™ EASY-Spray™ C18 HPLC Column, Catalog#ES902) using a linear gradient of LC-MS/MS grade acetonitrile (Sigma-Aldrich Inc.; 3–36%) in 0.1% formic acid. The elution duration was 110 min at a flow rate of 0.30 μL/min. Eluted peptides were ionized and sprayed into the mass spectrometer using a Nano-EasySpray Ion Source (Thermo Fisher Scientific) under the following settings: spray voltage, 1.6 kV; capillary temperature, 275 °C.

The Orbitrap Exploris 480 instrument was operated in data-dependent acquisition (DDA) mode to automatically switch between full-scan MS and MS/MS acquisition. Full-scan MS1 spectra were acquired with a resolution of 60,000, a scan range of *m*/*z* 300–1800, an AGC target of 1 × 10^5^, and a maximum injection time of 200 ms. The 15 most intense multiply charged ions (z ≥ 2) were sequentially isolated (isolation window of 2 *m*/*z*) and fragmented in the octopole collision cell by higher-energy collisional dissociation (HCD). Fragmentation was performed using a normalized collision energy of 30, an AGC target of 1 × 10^5^, a maximum injection time of 200 ms, and a resolution of 17,500. Dynamic exclusion was set to 20 s, and charge state screening was enabled to reject unassigned, 1+, and ≥7+ charge states.

Raw data files acquired from each sample were searched against the UniProtKB human protein sequence database (20,547 entries, downloaded 20 April 2020) and the NCBI SARS-CoV-2 protein sequence database (CovID19, 967 entries, downloaded 2 May 2023) using Proteome Discoverer 2.5 software (Thermo Scientific, San Jose, CA, USA) based on the SEQUEST algorithm and the Percolator PSM Validator. A maximum of two missed cleavages was allowed for the trypsin enzyme. Carbamidomethylation (+57.021 Da) of cysteines was specified as a fixed modification, while oxidation of methionine and deamidation of glutamine/asparagine (+0.98402 Da) were set as dynamic modifications. The minimum peptide length was constrained to five amino acids. The precursor mass tolerance was set to 15 ppm, and the fragment mass tolerance was set to 0.05 Da. The maximum false discovery rate (FDR) for peptides was specified as 0.01. The resulting Proteome Discoverer report contained all assembled proteins alongside their corresponding peptide sequences, peptide spectrum match (PSM) counts, and MS1 peak area abundance data. The mass spectrometry proteomics raw data have been deposited to the ProteomeXchange Consortium via the PRIDE partner repository with the dataset identifier PXD081034 and 10.6019/PXD081034.

### 2.7. Data Analysis (Bioinformatics/Statistical)

Only the high- and medium-confidence peptide matches with FDR ≤ 0.01 (high confidence) or FDR ≤ 0.05 (medium confidence—only few) were included in the data analysis described herein. The nano-LC/MS/MS database search identified the protein hits that are known to be associated with EVs based on the ExcoCarta open-source web-based repository [17]. Those proteins were considered as EV markers during the analysis. All the proteins associated with keratinocytes or skin cells, and some proteins potentially enriched from fetal bovine serum, were identified as contaminants and removed from the data analysis.

Due to the high individual heterogeneity, T0–T1 paired samples were analyzed to determine the fold-change of MS1 peak area abundance of each protein for each individual participant. T1 fold-change was calculated by dividing T1 peak area abundance by T0 (T1/T0) to identify proteins with increased (fold-change > 1) or decreased (fold-change < 1) abundance. Because our primary objective was to decode holistic molecular profiles and biological pathways rather than identify isolated, high-abundance biomarkers, all proteins demonstrating a differential abundance relative to their paired pre-infection baseline were included in STRING and Reactome analysis.

To analyze protein–protein interactions and functional enrichment, the uniport protein accession identifiers of both increased and decreased proteins of each cohort were pooled and searched using the “Homo sapiens-multiple search option” of the STRING v12.0 web version [18]. Unmapped proteins were automatically removed from the analysis, and mapped proteins were analyzed for “full STRING network” where the edges (connective lines) indicate both functional and physical protein associations. Line thickness of the network edges represents the confidence of protein interactions predicted by the strength of data supported from (1) text mining, (2) experiments, (3) databases, (4) co-expression, (5) neighborhood, (6) gene fusion and (7) co-occurrence [18]. The minimum required interaction score was set to high confidence (0.7). STRING nodes (proteins) were mapped to Reactome pathways and color-coded using the “Functional enrichment option” of the STRING v12.0. The protein–protein interaction (PPI) enrichment score reflects the statistical significance of the predictions.

To analyze downstream biological pathways and processes associated with increased and decreased proteins in each cohort, the uniport protein accession identifiers of each subset were uploaded to the Reactome pathway browser (v94). The over-representation analysis was carried out by Reactome against the human database without including the interactors to statistically determine (hypergeometric distribution) whether certain Reactome pathways are enriched in the submitted data [19].

Statistical analysis of the EV particle size/concentration and EV marker frequency comparisons was performed by two-tailed, paired *t*-test or, unpaired *t*-test with Welch’s correction using GraphPad Prism (v10.1.2, build 324), after testing the data for normality.

Heatmap and the bubble plots were generated using RStudio, v2024.12.1, build 563, and the pathway diagrams were created in BioRender (https://BioRender.com).

## 3. Results

### 3.1. PASC PBMC-EV Particles Are Smaller and Enriched with Exosomes and Micro-Vesicles

PBMCs are comprised of key immune cells, including lymphocytes (T cells, B cells, natural Killer cells) and monocytes, which actively secrete or scavenge EVs during intercellular communication. PBMC-EVs can be enriched ex vivo by allowing natural release of EVs from resting PBMCs to culture supernatant without exogenous stimulants. Ex vivo release of PBMC-EVs could depend on multiple factors, including individual variability in PBMC composition, cell viability and plating concentration. In the present study, we plated freeze-thawed PBMCs at 500,000 cells per well in 96-well tissue culture plates, incubated at 37 °C/5% CO_2_ for 22 h to allow natural EV release. EVs were enriched using ExoQuick Ultra columns following polymer-based precipitation of PBMC-EVs from culture supernatants (Figure 1A). The viability of freeze-thawed PBMC samples at the time of cell plating and harvesting after supernatant collection was high and consistent, with few exceptions (Table S2). In a few samples, post-harvesting viability was reduced, a phenomenon likely attributable to baseline inter-individual differences in PBMC subset composition, as all samples were processed identically (Table S2). There was no significant positive correlation between EV particle concentration analyzed by NTA and the PBMC viability (at cell plating or post-harvest) or the total number of PBMCs plated for EV enrichment (Table S2). The NTA data showed the presence of both exosomes and micro-vesicles in the enriched EV fractions with sizes ranging from 87 to 289 nm (Figure 1B). The smallest 10% particle fraction (D10) contained smaller particles between 87 and 130 nm in both PASC and non-PASC groups. The median particle size (50%) (D50) was significantly lower in the PASC individuals before the initial SARS-CoV-2 infection (T0) (143–160 nm) and at 6–8 months PASC survey (T1) (127–153 nm), compared to the non-PASC cohort (T0—149–198 nm, T1—144–186 nm). A similar trend was seen in the larger D90 EV particle fraction when less than 90% of the particles were considered but the difference did not reach statistical significance (Figure 1B). Particle concentration was statistically comparable across the PBMC-EVs enriched from PASC and non-PASC individuals (Figure 1C).

### 3.2. GAPDH Is a Frequent Marker Found in PBMC-EVs

**Figure 2:**
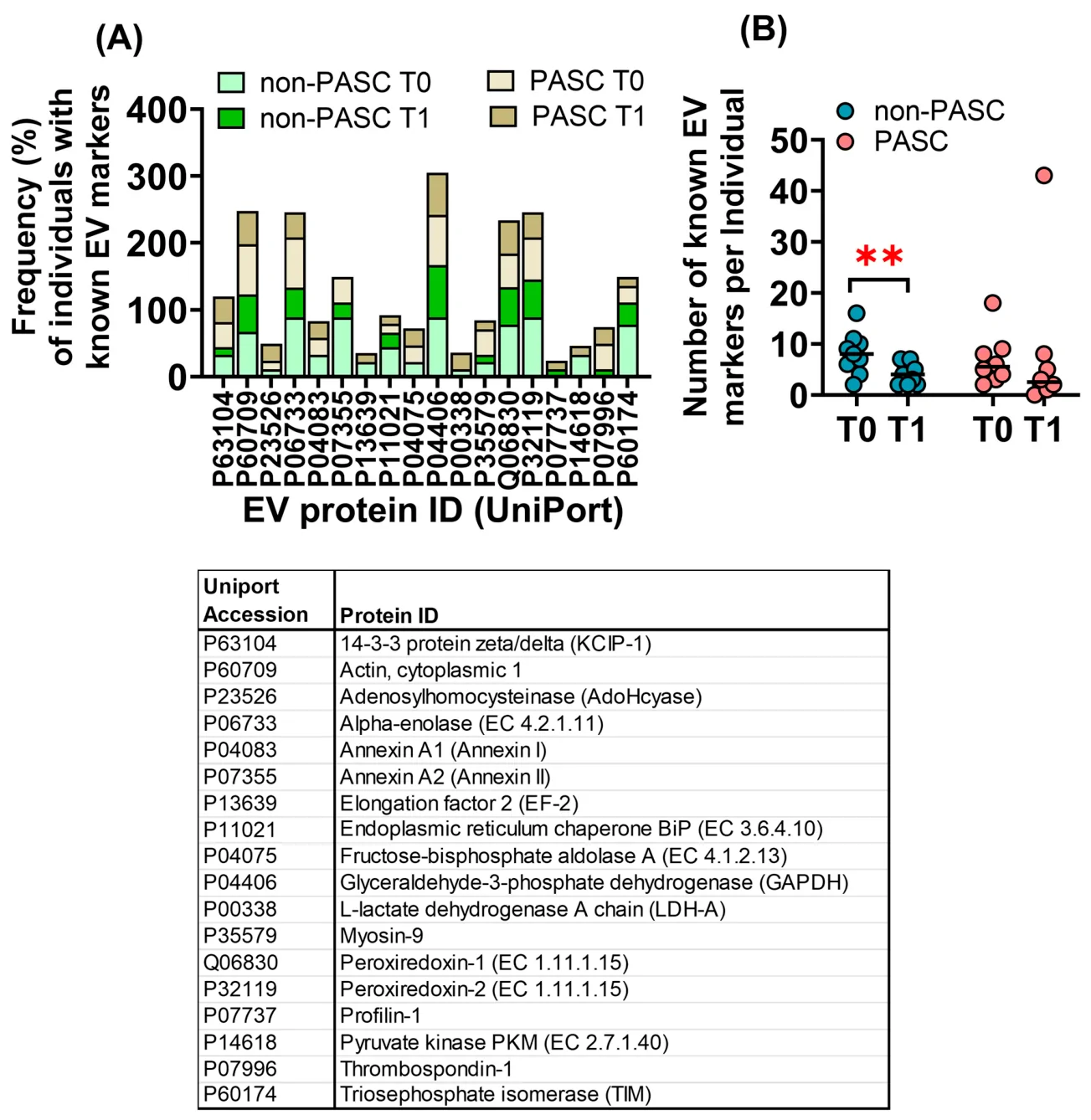
**PBMC-EV marker analysis**. (**A**) Frequency of PASC (*n* = 8) and non-PASC (*n* = 9) individuals expressing known EV markers matched against the ExoCarta database at T0 and T1 time points. Frequency of individuals with a given EV marker (detected by nano-LC-MS/MS) was calculated as follows: {Number of individuals with a given EV marker/Total number of individuals in the cohort (PASC or non-PASC at T0 or T1)} × 100. *Y*-axis shows the stacked frequencies of the four individual cohorts (each group representing 0–100%). *X* axis shows uniport accession IDs of the EV marker proteins matched against the ExoCarta database. Corresponding protein names are listed in the table. (**B**) Number of ExoCarta matched EV markers present in each individual. T0 (pre-infection) and T1 (6–8 months post-infection). Asterisks (*) denote ** *p* < 0.01 significance at 0.05 alpha level. Paired *t* test was used to compare between T0 and T1 after testing for normality.

Tetraspanins CD9, CD63 and CD81 have often been considered classical markers of exosomes or small EVs [20]. Tetraspanins represent a family of transmembrane proteins associated with plasma and endosomal membranes, mediating exosome secretion, uptake and cargo sorting into exosomes [20]. Emerging studies acknowledge that tetraspanins are heterogeneously distributed across different EV subpopulations and their expression may vary by the cellular source of the EVs [20,21]. Most of the published studies have used flow cytometry or Western blot-based methods to detect one or more tetraspanins to confirm the presence of EVs. Unfortunately, we could not validate these classical EV markers using a secondary validation method such as Western blot or flow cytometry, due to the small EV sample size as the availability T0 and T1 PBMCs were limited. Alternatively, in the present study, we compared PBMC-EV proteins identified by nano-LC-MS/MS against the ExoCarta database to confirm the presence of known EV markers in the enriched EV samples. We found glyceraldehyde 3-phosphate dehydrogenase (GAPDH) as the most frequently detected marker, alongside actin 1, α-enolase, annexin A2, peroxiredoxin-1, peroxiredoxin-2, and triosephosphate isomerase (TIM) across thirty-four PASC and non-PASC samples (Figure 2A). In fact, TIM, a crucial enzyme in glycolysis [22], was detected only in one PASC individual (with headache and drowsiness) out of eight (12.5%) at T1, while it was also lower at T0 in PASC (25%) compared to non-PASC (Figure 2A). In non-PASC, 78% of the individuals had TIM at T0, while it was reduced to 33% by the T1 PASC survey (Figure 2A). CD9 was detected only in one sample (out of 34 individual samples derived from paired PBMC samples), indicating that the detection of unevenly distributed tetraspanins may be obscured by the relative enrichment of many other proteins detected by nano-LC-MS/MS. We did not detect CD63 or CD81 by nano-LC-MS/MS. In both PASC and non-PASC individuals, the number of known EV markers present in each individual was lower at 6–8 months post-SARS-CoV-2 infection compared to the pre-infection, where it was statistically significant in non-PASC (Figure 2B). One PASC participant (PASC8) failed to meet the quality-control threshold due to the absence of detectable ExoCarta-annotated EV markers at the T1 time point. Therefore, PASC8 T0 and T1 samples were excluded from downstream STRING and Reactome pathway analysis.

### 3.3. Nano-LC/MS/MS Analysis Show Distinct PBMC-EV Proteomic Signatures 6–8 Months Post-Infection

To analyze PBMC-EV proteome dynamics of each PASC and non-PASC individual, we first calculated the fold-change of abundance of each protein at T1 relative to T0. Proteins with missing values in either T0 or T1 were excluded to allow for precise intra-individual fold-change calculation (T1/T0). Given the high individual heterogeneity and small cohort size, we deliberately avoided data imputation. While missing values often occur due to several reasons such as true absence, individual heterogeneity, biological variations or technical sensitivity limits, imputing them would introduce artificial variance that could disproportionately skew the resulting fold-changes.

**Figure 3:**
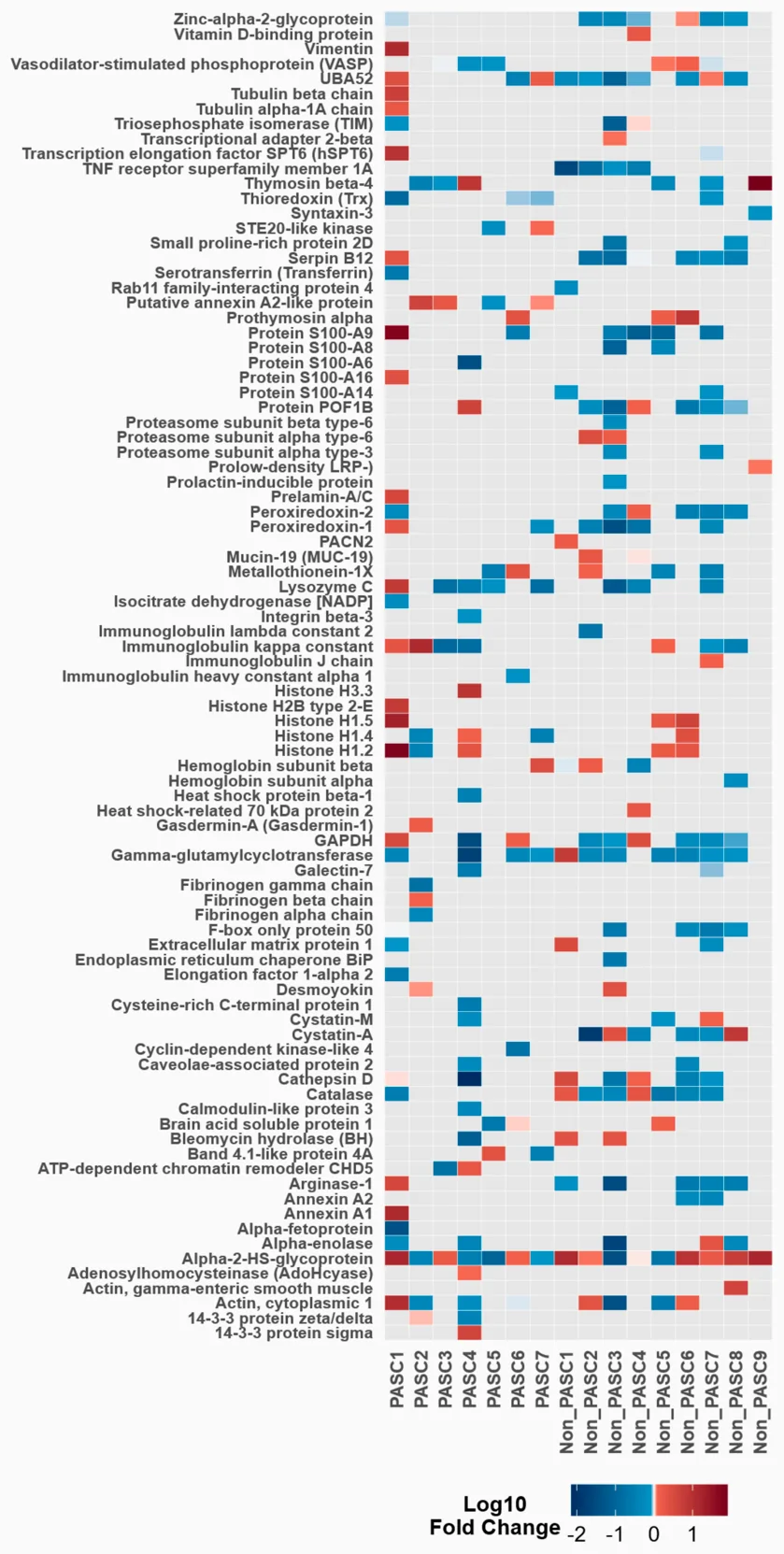
**T1 fold-change abundance of EV proteins imprinted by different PASC symptoms.** Fold-change heatmap illustrates PBMC-EV proteins with increased or decreased T1 abundance in PASC (*n* = 7, 1–7) and non-PASC (*n* = 9, 1–9) individuals. Protein names are displayed in the Y axis. PASC1—headache and drowsiness, PASC2–6—pulmonary complications, PASC7—loss of smell. Protein abundance (MS1 peak) was used to calculate fold-change between T0 (pre-infection) and T1 (6–8 months post-infection). Fold-change > 1 = increased abundance (shades of red); fold-change < 1 = decreased abundance (shades of blue). Fold-change values were log_10_-transformed.

Protein abundance was considered to be increased or decreased at T1 based on the fold-change (Figure 3, Table S3). We further analyzed PASC individuals by their history of prolonged PASC symptoms (>30 days) as follows: (1) headache and drowsiness, (2) pulmonary complications and (3) loss of smell. Only one individual reported a history of headache and drowsiness (PASC1), and one had a history of loss of smell (PASC7), which were considered as case observations (Table S1). The five individuals who had a history of one or more prior pulmonary PASC symptoms were included in a single subgroup (PASC2–PASC6) (Table S1). In the pulmonary PASC individuals, the same protein was differentially abundant across the individuals or absent in most cases. Therefore, the data were pooled to analyze cohort-level protein interactions and biological pathways. The PBMC-EV proteome revealed a diverse landscape of cohort-specific changes capturing inherent individual heterogeneities (Figure 3, Table S3). The heatmap shows the distribution of PBMC-EV proteins with increased (↑) and decreased (↓) abundance by T1 across the individuals in both PASC and non-PASC cohorts (Figure 3).

Nano-LC-MS/MS generated 169 protein hits in PASC1 T0 and >500 in T1. Meanwhile for PASC2-7, T0 protein hits ranged from 93 to 316, and T1 analysis resulted between 76 and 214 hits. The data represent the number of MS hits before excluding low FDR hits (<0.05) and potential contaminants (skin proteins and contaminants from fetal bovine serum). In addition, non-EV proteins such as calnexin and GRP94 were not found among the resulting MS hits across 34 samples. Overall, PASC1 (headache and drowsiness) showed 21 increased and 13 decreased proteins, while the pulmonary PASC had 19 increased proteins and 36 decreased proteins (cohort level) (Figure 3, Table S3). In PASC1 headache and drowsiness, nuclear and cytoskeleton proteins were highly enriched in the PBMC-EV proteome. Particularly, linker histone H1.2, was increased by 66-fold and H1.5 by 18-fold; meanwhile, core histone H2B type 2-E, transcription elongation factor SPT6 and prelamin A/C also showed a substantial increase (Figure 3, Table S3). Cytoskeleton proteins including actin, 1 cytoplasmic, vimentin and the tubulin chains, alpha 1A (α1A) and beta (β) also showed increased abundance in PASC1. In addition, one of the alarmin proteins, S100-A9, was increased by 56-fold. Several proteins including arginase-1, lysozyme C and α-2-HS-glycoprotein associated with neutrophil degranulation and cellular damage also showed increased abundance (Figure 3, Table S3). Abundance of those proteins associated with glycolysis/gluconeogenesis (TIM and alpha(α)-enolase) and redox balance (peroxiredoxin-2 and thioredoxin) was markedly decreased in PASC1 (Figure 3, Table S3). Pulmonary PASC had a characteristic increase in histone H3.3 (8-fold) and differential abundance of 14-3-3 isoforms (ζ—zeta/δ—delta and σ—sigma) with a 4-fold increase of 14-3-3 σ in one individual. Differential abundance of fibrinogen chains was also notable in one of the pulmonary PASC individuals (Figure 3, Table S3). Loss of smell was associated with several increased proteins including STE20-like protein (Figure 3, Table S3). The non-PASC cohort had the highest number of proteins with increased (37 proteins) or decreased (44 proteins) T1 abundance at the cohort level among all the four groups. Notably, both gamma (ɣ)-glutamylcyclotransferase and thioredoxin showed decreased abundance in at least one individual across each cohort, indicating common biological responses. Likewise, several proteins including GAPDH, α-enolase, actin, 1, histone H1.2, ubiquitin ribosomal protein eL40 fusion protein (UBA52), lysozyme C and S100-A9 were common to both PASC1 headache and drowsiness individual and some of the pulmonary PASC individuals, even though their fold-change abundance was different (Figure 3, Table S3). Our data highlight the heterogeneity and overlap of molecular alterations across different PASC symptom presentations and non-PASC individuals.

### 3.4. STRING and Pathway Enrichment Analysis Identifies Distinct Biological Signatures in PBMC-EV Proteome

**Figure 4:**
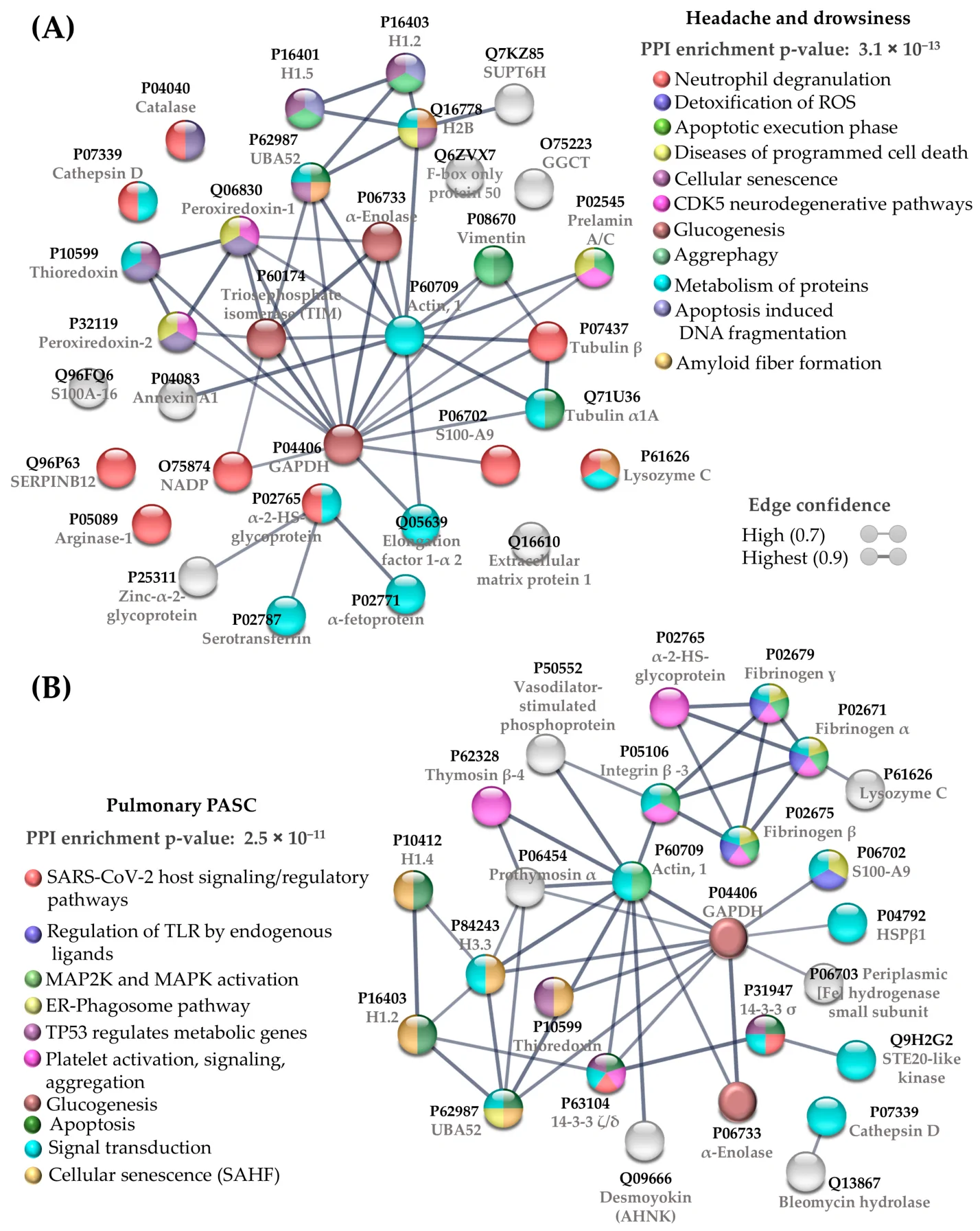
**STRING protein interactions and functional enrichment analysis of PBMC-EV proteins with increased and decreased T1 abundance in PASC individuals**. (**A**) headache and drowsiness (*n* = 1), (**B**) pulmonary PASC (*n* = 5). Nodes = Uniport accession IDs and names of the proteins, color-coded by functionally enriched Reactome pathways. Disconnected proteins are hidden in (**B**).

To examine functional relationships among dysregulated proteins in individuals with different PASC symptoms, we first constructed STRING protein interaction networks, which were automatically mapped to major Reactome pathways filtered by an FDR cut-off of <0.05 (Figure 4). Protein hits from the individual who reported headache and drowsiness as well as the pooled protein hits from the five pulmonary PASC participants formed significantly enriched protein networks (PPI enrichment *p*-value 3.1 × 10^−13^ for headache/drowsiness and 2.5 × 10^−11^ for pulmonary PASC), indicating non-random interactions and coordinated biological functions that persisted long after acute infection (Figure 4). The individual with a history of PASC-related loss of smell did not yield a meaningful network, likely due to the small number of dysregulated protein hits detected in the enriched EV preparation. To identify significant downstream biological pathways, we performed an independent and comprehensive Reactome pathway analysis using UniProt accession identifiers of proteins showing increased or decreased abundance. The significantly enriched pathways associated with increased or decreased T1 abundance, reflecting potential biological activation or suppression, were filtered by the FDR < 0.05 and the number of mapped proteins ≥ 2 (Figures S1 and S2). The single cases with a history of PASC headache–drowsiness or the loss of smell were treated as case observations. For pulmonary complications, pathways resulting from proteins with decreased T1 abundance were filtered by FDR cut-off <0.01 to enhance the stringency due to the increased number of enriched pathways at FDR < 0.05 cut-off (Figure S2).

#### 3.4.1. STRING Network Analysis of the Individual with a History of PASC Headache and Drowsiness—A Case Observation

The PBMC-EV proteome of PASC1 headache and drowsiness revealed a highly interconnected network centered on four proteins: TIM↓, α-enolase↓, GAPDH↑, and actin, cytoplasmic 1↑ (Figure 4A, Table S3). The glycolytic enzymes, TIM, α-enolase and GAPDH, were mapped to gluconeogenesis and glycolysis pathways. Meanwhile, actin, cytoplasmic 1, the key cytoskeleton protein, was mapped to protein metabolism. Together, this metabolic–structural protein hub integrated four major functional modules involving (1) redox imbalance and oxidative stress, (2) chromatin remodeling, (3) cytoskeletal remodeling, and (4) immune-metabolic stress and chronic inflammation.

Redox imbalance and oxidative stress were represented by a highly interactive cluster of redox-regulating proteins: peroxiredoxin 1↑, peroxiredoxin 2↓ and thioredoxin↓ (Figure 4A, Table S3) (with medium confidence [0.4] associations to catalase↓ and cathepsin D↑). The proteins were mapped to detoxification of reactive oxygen species (ROS), diseases of programmed cell death, cyclin-dependent kinase 5 (CDK5)-related neurodegenerative diseases, neutrophil degranulation, and protein metabolism. The redox protein nodes were directly linked to the metabolic–structural hub, suggesting that oxidative stress is closely coupled with metabolic and cytoskeletal stress responses.

The second major module consisted of histones involved in chromatin remodeling including histone H2B type 2-E↑, histone H1.5↑, and histone H1.2↑ (Figure 4A, Table S3). The linker histones H1.2 and H1.5 were associated with apoptosis and cellular senescence pathways. UBA52↑ served as a key node linking between chromatin and cytoskeleton remodeling. Histone H2B type 2-E and UBA52 were mapped to protein metabolism, aggrephagy, amyloid fiber formation, and cellular senescence.

The cytoskeletal structural protein module contained tubulin α-1A chain↑, tubulin β chain↑, vimentin↑, and prelamin A/C↑ converging into the central metabolic–structural triad through actin, cytoplasmic 1↑ (Figure 4A, Table S3). The proteins in this module were mapped to protein metabolism, aggrephagy, neutrophil degranulation, apoptotic execution phase and CDK5 neurodegenerative pathways (Figure 4A).

The cluster centered on GAPDH↑ was positioned at the interface of these functional modules, co-annotated with proteins associated with neutrophil degranulation (S100-A9↑ and isocitrate dehydrogenase [NADP]↓) and protein metabolism (elongation factor 1-α 2↓) (Figure 4A). In addition, the STRING analysis at medium confidence (0.4) showed that this cluster is weakly associated with additional proteins involved in neutrophil degranulation, cellular damage (arginase-1↑, lysozyme C↑, α-2-HS-glycoprotein/Fetuin-A↑), and protein metabolism (α-fetoprotein↓, and serotransferrin↓).

#### 3.4.2. STRING Network Analysis of Pulmonary PASC Suggests Coordinated Alterations in Cell Fate Decisions, Platelet Function and Immune Signaling Pathways

Here, we present cohort-level functional protein interactions in pulmonary PASC, where we pooled protein hits from five individuals showing increased and decreased fold-change abundance. Differentially abundant proteins were present in only one or a subset of individuals in this cohort, as previously shown in Figure 3 (PASC2-6).

The STRING network was marked by four interconnected metabolic–redox-structural proteins composed of GAPDH↑↓, α-enolase↓, thioredoxin↓, actin, cytoplasmic 1↓ and 14-3-3 ζ/δ↑/↓ (Figure 4B, Table S3). The first major cluster contained histone proteins associated with chromatin organization: histone H1.2↑/↓, histone H1.4↑/↓, histone H3.3↑ and UBA52↓ linked to GAPDH↑↓, 14-3-3 ζ/δ↑/↓ and actin, cytoplasmic 1↓ (Figure 4B, Table S3). The histones were mapped to apoptosis, cellular senescence and signal transduction (Figure 4B). 14-3-3 ζ/δ↑/↓ was mapped to multiple functions including TP53-mediated metabolic regulation, signal transduction, apoptosis, platelet activation, and SARS-CoV-2 host signaling pathways (Figure 4B, Table S3). The data suggest a potential association between metabolic-redox-viral stress and signaling pathways regulating cell fate decisions.

The second major cluster was characterized by thymosin β-4↑/↓, vasodilator-stimulated phosphoprotein↓ and integrin β-3↓ radiating from actin, cytoplasmic 1↓ (Figure 4B, Table S3). These proteins are known to be associated with cytoskeletal dynamics, calcium signaling, and platelet activation/signaling/aggregation. Functional enrichment mapped actin, 1 to mitogen-activated protein kinase (MAP2K/MAPK) signaling and thymosin β-4 to platelet activation process. Integrin β-3↓ showed strong connections to a fibrinogen-centered submodule composed of fibrinogen β chain↑, fibrinogen ɣ chain↓, fibrinogen α chain↓, α-2-HS-glycoprotein↑/↓, and lysozyme C↓ (Figure 4B, Table S3). Protein S100-A9↓ was weakly associated with this cluster at medium confidence (0.4) cut-off. These proteins were mapped to platelet activation/signaling/aggregation, MAPK2/MAPK signaling, regulation of Toll-like receptors and the ER–phagosome pathways, suggesting coordinated disturbances in platelet function and immune signaling.

#### 3.4.3. Pathway Enrichment Analysis of the Individual with a History of PASC Headache and Drowsiness—A Case Observation

**Figure 5:**
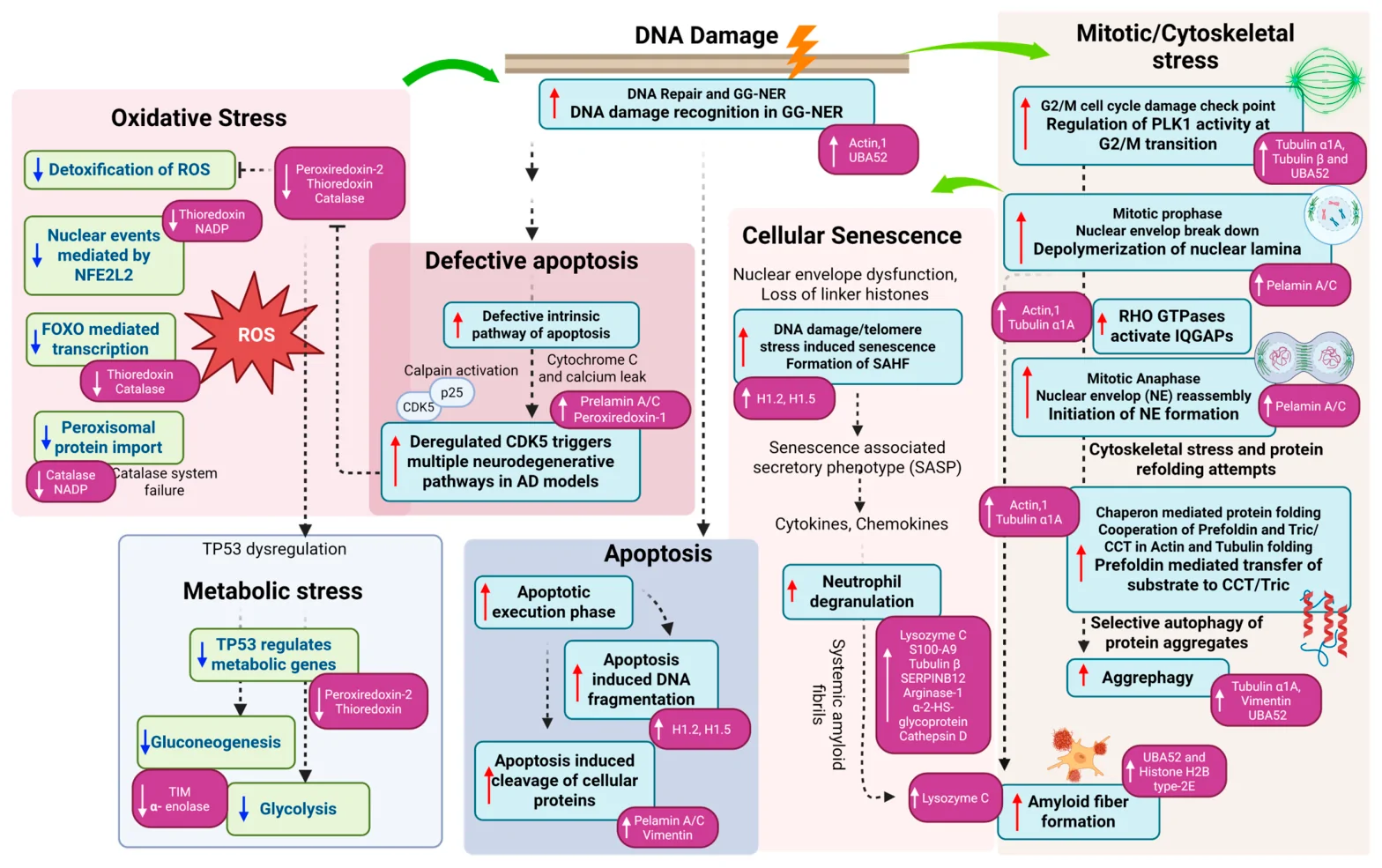
**Pathway enrichment analysis of the individual with a history of PASC headache and drowsiness.** Significantly enriched, downstream Reactome pathways mapped to nano-LC-MS/MS protein hits with increased T1 abundance are represented by light blue boxes with red upward arrows and decreased T1 abundance are represented by light green boxes with blue downward arrows. Major proteins mapped to the enriched pathways are indicated by orchid color (purple shade) boxes with white upward (increased T1) or downward (decreased T1) arrows.

After mapping 34 PBMC-EV proteins with differential fold-change abundances, we identified significantly enriched Reactome pathways with biological relevance in the individual with a history of PASC headache and drowsiness (Figures S1 and S2 and Figure 5). The data are presented as a single case observation.

Oxidative stress was annotated to decreased peroxiredoxin-2/thioredoxin levels and antioxidant pathways (Figure 5 and Figure S2). Increased levels of actin, cytoplasmic 1 and UBA52 in PBMC-EVs were enriched with DNA damage recognition and repair pathways (Figure 5 and Figure S1, Table S3). Both actin and UBA52 have shown to be involved in DNA repair and apoptosis [23,24,25]. Increased levels of prelamin A/C and peroxiredoxin-1 were enriched with “deregulated CDK5 pathway triggers multiple neurodegenerative pathways in Alzheimer’s Disease (AD) models” pathway suggesting defective apoptosis (Figure 5 and Figure S1, Table S3). Decreased thioredoxin (0.125-fold) and peroxiredoxin-2 (0.546-fold) were enriched with TP53-dependent regulation of metabolic genes pathway. Subsequently, gluconeogenesis and glycolysis were mapped to decreased levels of TIM (0.714-fold) and α-enolase (0.588-fold) (Figure 5 and Figure S2, Table S3).

Concurrently, the PBMC-EV proteome was enriched with pathways associated with mitotic stress, nuclear envelope dysfunction, and cytoskeletal stress (Figure 5). Regulation of polo-like kinase 1 (PLK1) activity at the G2/M transition was mapped to increased levels of UBA52, tubulin α1A and β chains (Figure 5 and Figure S1, Table S3), indicating dysregulation of PLK1-mediated spindle organization, microtubule dynamics, and kinetochore function during mitosis [26]. In addition, nuclear envelope breakdown and reassembly pathways were mapped to increased accumulation of lamin A precursor, prelamin A/C (3.524-fold) in PBMC-EVs (Figure 5 and Figure S1, Table S3). Lamin A is the processed form of prelamin A/C, a key component of the nuclear lamina supporting the nuclear membrane to prevent mechanical stress [27]. During open mitosis, phosphorylation of both A and B type lamins is essential for nuclear envelope breakdown, enabling microtubule access to the chromosomes [27]. Dephosphorylated lamins must be reassembled into nuclear lamina to support the newly reformed nuclear envelope during mitotic exit *vice versa* [27].

Increased levels of microtubules (tubulin chains), actin, cytoplasmic 1, and vimentin were enriched with Reactome pathways associated with protein folding and aggrephagy (Figure 5 and Figure S1, Table S3). Mechanistically, intermediate vimentin filaments must be localized to the actin cortex (attached to the inner layer of the plasma membrane) during mitosis to complete cell division [28]. Dysfunctional or dysregulated vimentin could impair mitotic exit and cell division inducing mitotic stress [28]. Enriched pathways related to actin 1 and tubulin refolding attempts suggest subsequent proteostasis stress leading to protein misfolding and aggrephagy (Figure 5 and Figure S1, Table S3). 

Pathway analysis also showed that high levels of free H1.2 and H1.5 linker histones found in PBMC-EVs (Table S3) were mapped to DNA damage/telomere stress-induced formation of senescence-associated heterochromatin foci (SAHF) pathway (Figure 5 and Figure S1). SAHF are a defining feature of senescent cells, particularly formed in response to stress signals. Senescent cells develop senescence-associated secretory phenotype (SASP) and secrete a diverse array of pro-inflammatory mediators. As a result, SASP can trigger low grade inflammation leading to chronic neutrophil degranulation. Our nano-LC/MS/MS analysis detected seven proteins associated with neutrophil degranulation pathway present at high levels in PBMC-EVs of PASC1. These included the proteins found in tertiary/gelatinase granules (cathepsin D, arginase-1, S100-A9, lysozyme C), secondary/specific granules (lysozyme C, S100-A9), and primary granules (lysozyme C) (Figure 5 and Figure S1, Table S3). Our dataset was also enriched with amyloid-fiber formation pathway mapped to highly abundant UBA52, core histone H2B type 2-E and lysozyme C (Table S3, Figure S1 and Figure 5).

These data suggest that PBMC-EVs in the PASC1 individual with a history of PASC headache and drowsiness carry stress signals lingering up to 6–8 months post-infection.

#### 3.4.4. PBMC-EVs of Pulmonary PASC Are Enriched with Cell Fate, Platelet and Immune-Related Pathways

Similar to PASC1 headache and drowsiness, we comprehensively analyzed significantly enriched downstream Reactome pathways of those five individuals (PASC2-6) with a history of Pulmonary PASC. As mentioned earlier, the protein hits from PASC2-6 were pooled due to the high EV proteome heterogeneity.

**Figure 6:**
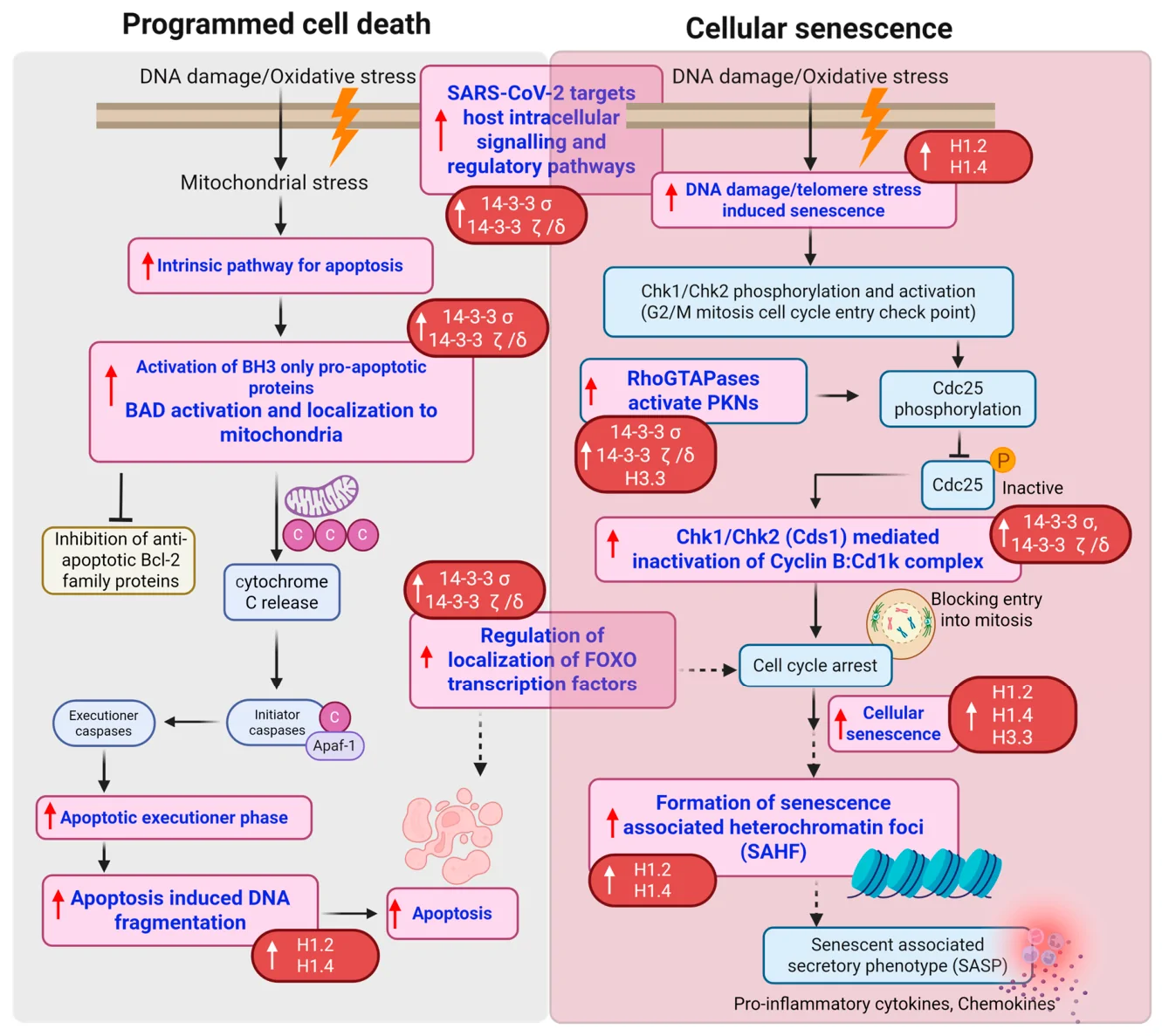
**Reactome pathway enrichment analysis of the individuals with a history of pulmonary PASC suggesting alterations in cell fate decisions**. Nano-LC-MS/MS proteins hits from five individuals were pooled during the analysis. Significantly enriched, downstream Reactome pathways mapped to the proteins with increased T1 abundance are represented by pink boxes with red upward arrows. Major proteins mapped to enriched pathways are indicated by dark red color boxes with white upward arrows. C—cytochrome C, P—phosphorylation.

The pooled analysis showed enriched downstream Reactome pathways linked to apoptotic and senescence cell fate decisions (Figure 6 and Figure S1). PBMC-EVs from one of the pulmonary PASC participants had increased levels of H1.2 and H1.4 histones, enriched with DNA damage or telomere stress induced senescence pathways (Figure 6, Table S3). While viral persistence could be a main source of chronic DNA damage response, we did not detect SARS-CoV-2 proteins in the PBMC-EV proteome. However, increased abundance of 14-3-3 isoforms was associated with host intracellular signaling/regulatory pathways targeted by SARS-CoV-2 (Figure 6 and Figure S1, Table S3). 14-3-3 isoforms were also mapped to activation of Bcl-2 homology 3 (BH3)-only pro-apoptotic proteins and Bcl-2-associated death promoter (BAD) localization to mitochondria pathways indicating mitochondrial stress and apoptosis. Downstream apoptosis pathways were enriched by elevated linker histones H1.2 and H1.4 (Figure 6 and Figure S1, Table S3). In parallel, checkpoint kinase 1/2 (Chk1/Chk2)-mediated inactivation of Cyclin B–Cdk1 complex pathway was mapped to increased 14-3-3 levels, suggesting cell cycle arrest (Figure 6 and Figure S1). Our data further implied that 14-3-3 may also interfere with pathways related to regulation of localization of FOXO transcription factors modulating apoptosis and cellular senescence (Figure 6 and Figure S1). We also found high levels of free histone H3.3 (8.2-fold), along with H1.2 and H1.4 in the EV proteome mapped to cellular senescence and SAHF pathways (Table S3, Figure S1 and Figure 6).

**Figure 7:**
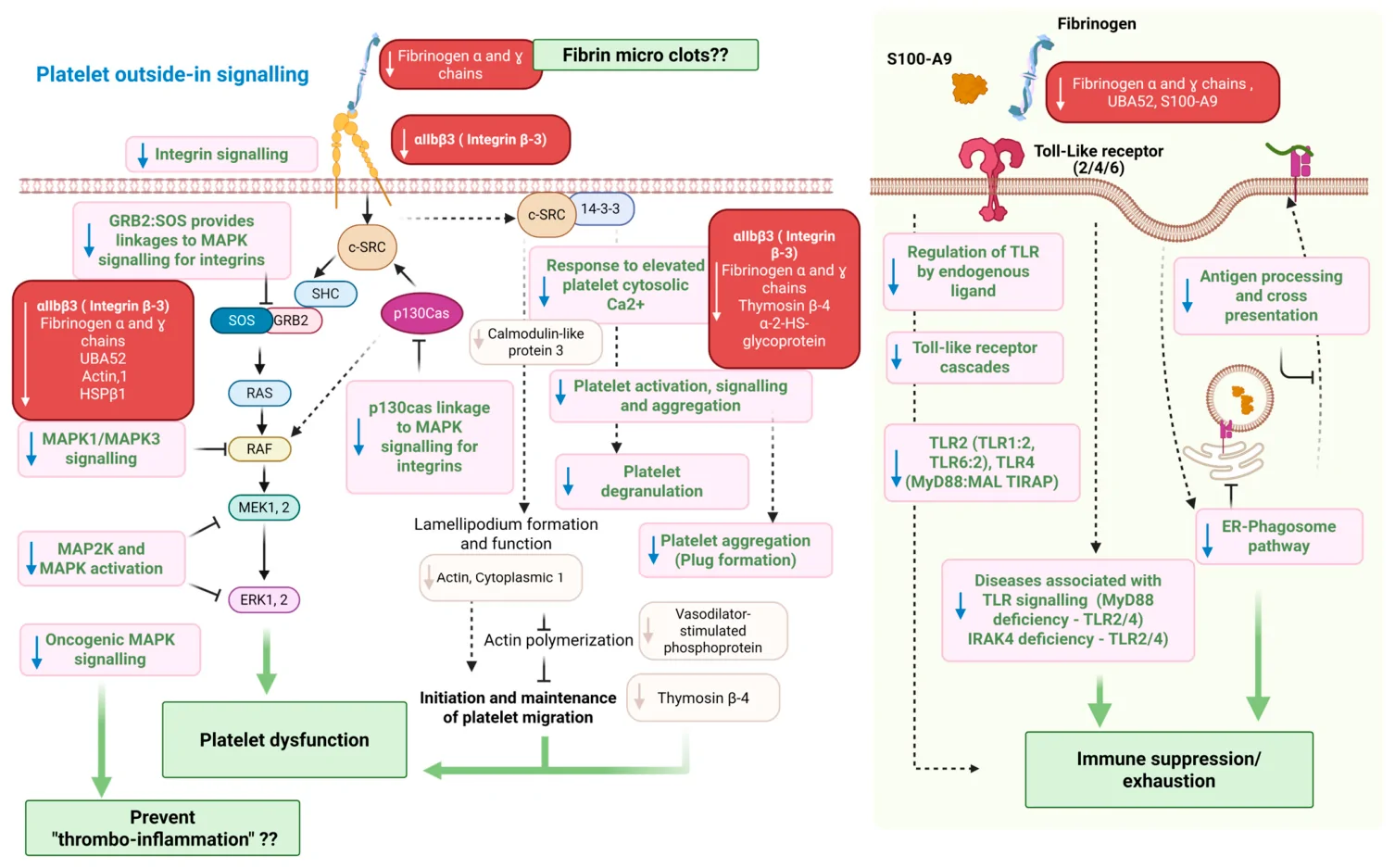
**Reactome pathway enrichment analysis of the individuals with a history of pulmonary PASC suggesting altered platelet function and immune signaling**. Nano-LC-MS/MS protein hits from five individuals were pooled during the analysis. Downstream Reactome pathways enriched with proteins showing decreased T1 abundance are represented by light pink boxes and blue downward arrows. Major proteins mapped to enriched pathways are indicated by dark red color boxes with white downward arrows. Other proteins found with decreased T1 abundance in PBMC-EVs, potentially associated with the pathways are indicated by pale-colored boxes with downward arrows.

Conversely, PBMC-EVs had decreased T1 levels of integrin β-3 (0.69-fold) and the two fibrinogen chains (α, 0.468-fold and ɣ, 0.171-fold) contributing to the enrichment of platelet outside–in signaling pathways (Figure 7 and Figure S2, Table S3). This was observed in the pooled data where one individual (PASC4) had decreased integrin β-3 and the other had decreased fibrinogen chains (PASC2) (Table S3). When closely analyzed, we found high levels of integrin β-3 in T0, with a missing value at T1 in PASC2. Likewise, the nano-LC/MS/MS detected fibrinogen ɣ chain at a high confidence (FDR ≤ 0.01) at T0 in PASC4, but did not produce quantifiable MS1 peak at T1, resulting in a missing value. Decreased levels of fibrinogen and integrin β-3 proteins were associated with the enrichment of regular and oncogenic MAPK signaling (Ras-Raf–MEK–ERK) through GRB2: SOS or p130cas engagement pathways (Figure 7 and Figure S2). Furthermore, decreased T1 abundance of calmodulin-like protein 3 in the PBMC-EVs (Table S3) suggests altered Ca^2+^ signal transduction. Additionally, we found decreased levels of actin, cytoplasmic 1, vasodilator-stimulated phosphoprotein, and thymosin β-4 in the pulmonary PASC cohort (Table S3). Reactome analysis enriched pathways related to cytosolic Ca^2+^ responsiveness, platelet activation, platelet degranulation, and aggregation involving some of these proteins (Figure 7 and Figure S2).

Concomitantly, our data were enriched with toll-like receptor (TLR) and antigen cross-presentation pathways (Figure S2 and Figure 7). All these downstream pathways had three common proteins mapped with decreased T1 abundance: fibrinogen (α and ɣ chains) and S100-A9 (Table S3, Figure 7). The pathways included regulation of TLR by endogenous ligands and canonical TLR signaling cascades (TLR2/4/6). This was accompanied by the enrichment of pathways associated with MyD88 and IRAK4 deficiency (Figure S2 and Figure 7). In parallel, decreased fibrinogen chains, S100-A9 and UBA52, were associated with antigen processing and cross-presentation pathways through endoplasmic reticulum (ER)–phagosome axis (Figure S2 and Figure 7).

Collectively, our results suggest that apoptosis, senescence, platelet and immune signaling pathways could remain altered at 6–8 months post-infection in the five individuals who reported to have a history of pulmonary PASC.

#### 3.4.5. STRING and Reactome Analysis of Non-PASC Individuals Show a Heterogeneous Spectrum of Damage Resolution, with Signs of Cellular Stress and Chronic Inflammation

For the non-PASC cohort, individual-level pathway analysis was carried out on their EV-PBMC proteins showing increased or decreased T1 abundance (Table S4, Figures S1 and S2). The significant and most terminal pathways from each individual were sorted and visualized by the bubble plots (Figures S1 and S2). Non-PASC8 did not yield any statistically significant pathway enrichment; meanwhile, non-PASC2 and 9 did not result in any enriched pathways mapped to proteins showing decreased abundance to satisfy the statistical cut-offs (FDR < 0.05). In addition, non-PASC9 was only enriched with platelet degranulation showing 79-fold increase in thymosin-β. The remaining individuals showed overlapping, yet individual-specific physiological trajectories 6–8 months following SARS-CoV-2 infection (Figures S1 and S2, Tables S3 and S4).

Pathway analysis of the two non-PASC individuals (non-PASC1 and 4) indicated a coordinated “immune and metabolic resolution”. Non-PASC1 and 4 were enriched with neutrophil degranulation pathways mapped to elevated levels of buffering agents, including catalase (antioxidant), cathepsin D (regulates neutrophil apoptosis), and α-2-HS-glycoprotein (anti-inflammatory) [29] (Figure S1, Tables S3 and S4). Conversely, decreased alarmin S100-A9, anti-microbial peptide lysozyme C (non-PASC4), and T cell suppressor arginase-1 (non-PASC1) were also associated with neutrophil degranulation pathway (Figure S2, Tables S3 and S4). In non-PASC4, this protective neutrophil buffering was coupled with enriched glycolysis/gluconeogenesis (GAPDH↑, TIM↑) and ROS detoxification pathways (peroxiredoxin-2↑, catalase↑) (Figure S1, Tables S3 and S4). Also, non-PASC4 showed decreased peroxiredoxin-1 and UBA52 levels mapped to transcriptional regulation by TP53 and nuclear events mediated by NFE2L2 suggesting incomplete resolution of metabolic and oxidative stress (Figure S2, Tables S3 and S4). In addition, decreased levels of lysozyme C were mapped to amyloid fiber formation in non-PASC4. Autophagy was enriched with decreased abundance of UBA52 and hemoglobin subunit β in non-PASC1 and 4 (Figure S2, Tables S3 and S4). Concurrently, several other decreased T1 proteins were associated with master cell death/inflammatory regulators (TNFR1/TNFR2 NF-κB pathways; non-PASC1 and 4), interleukin signaling (non-PASC1 and 4), TLR cascades (TLR1/2/4/6; non-PASC4), and ER-phagosome pathways (non-PASC4) in these two individuals (Figure S2, Tables S3 and S4).

More than two-fold increase in actin, cytoplasmic 1 in non-PASC2 PBMC-EV proteome suggests unresolved cytoskeleton stress, even though we did not find any proteins/pathways associated with nuclear remodeling as seen with PASC individuals (Tables S3 and S4). However, unlike non-PASC1 and 4, non-PASC2 was enriched with UCH proteinases pathway mapped to increased levels of proteasome subunit α type-6 suggesting active protein degradation (Figure S1, Tables S3 and S4). In addition, elevated mucin-19 and proteasome subunit α type-6 were mapped to enriched C-type lectin receptor (CLR) pathways in non-PASC2, suggesting increased recognition of mucin-19 by CLRs and increased proteasomal degradation of mucin-19 (Figure S1, Tables S3 and S4). Mucin-19 expression appears to be induced by respiratory viruses [30], and increased levels of mucins have been found after SARS-CoV-2 infection [31]. Non-PASC2 also showed signs of non-pathogenic neutrophil degranulation in the absence of inflammatory mediators such as S100-A9 and lysozyme C (Figure S1, Tables S3 and S4).

In non-PASC3, neutrophil degranulation pathway was mapped to decreased alarmins (S100-A8/A9↓) and antioxidant/anti-inflammatory molecules (cathepsin D↓, catalase↓, and SerpinB12↓). Subsequently, reduced levels of S100-A8/A9 suggested persistent alterations in ER-phagosome-antigen cross-presentation pathway (Tables S3 and S4). Nevertheless, increased abundance of bleomycin hydrolase, a peptide trimmer in the MHC-class I processing pathway suggests attempts in active class I MHC-mediated antigen processing (Tables S3 and S4). Non-PASC3 also shows signs of immunosuppression through enriched non-canonical TNFR2 NF-κB pathways mapped to decreased TNF1A protein, UBA52 and proteosome subunits (Tables S3 and S4). Suppressed NFE2L2-mediated events mapped to decreased peroxiredoxin-1, and the proteasome subunits α type-3 and β type-6 may indicate a lingering vulnerability to oxidative stress (Tables S3 and S4). Unlike non-PASC2, decreased actin, cytoplasmic 1 was mapped to CDH1 function regulation and MAPK cascades cytoskeletal remodeling (Tables S3 and S4). Energy metabolism signatures appeared to be altered in non-PASC3, marked by decreased abundance of GAPDH, α-enolase, TIM, and arginase-1 (Tables S3 and S4). Enriched Ub-specific processing proteases pathway mapped to transcriptional adapter 2-β↑ and proteasome subunit α type-6↑ correlates with the pathways supporting protein metabolism (Tables S3 and S4). Noticeably, the proteasome subunit proteins (α type-6, α type-3, and β type-6) in the ubiquitin-proteasome system were highly dysregulated in non-PASC3, which may reflect persistent molecular alterations in the post-acute phase of the SARS-CoV-2 infection (Tables S3 and S4). Overall, the non-PASC3 PBMC-EV proteome signature showed a state of active resolution of inflammation and metabolism while attempting to augment MHC-1 antigen processing.

Across non-PASC individuals 5, 6, and 7, there was a marked decrease in the T1 abundance of proteins associated with neutrophil degranulation (Tables S3 and S4). Similar to pulmonary PASC, non-PASC5 and 6 exhibited enriched apoptosis and senescence linked to elevated linker histones (H1.2, H1.4, and H1.5) (Tables S3 and S4). PBMC post-thaw and post-EV harvest viability was high in non-PASC6, whereas non-PASC5 PBMCs had lower viability at post-thaw and further decreased after the EV harvest (Table S2). However, we excluded excessive cell death as an incriminating factor responsible for the EV histones in these individuals, as PASC1, 2 and 4, who had consistently high PBMC viability, also had high levels of histones in PBMC-EVs (Tables S2 and S3). Meanwhile, decreased S100-A8/A9 levels were mapped to TLR cascades and the ER-phagosome pathway in non-PASC5 (Tables S3 and S4). Likewise, platelet degranulation was associated with decreased thymosin β-4 and α-2-HS-glycoprotein in non-PASC5 (Tables S3 and S4). The enriched extracellular matrix interactions driven by increased levels of vasodilator-stimulated phosphoprotein and actin, cytoplasmic 1 suggest efforts to restore platelet morphology and function in non-PASC6 (Tables S3 and S4). Non-PASC7 was enriched with ROS detoxification and TP53-mediated metabolic gene regulation pathways mapped to decreased levels of peroxiredoxin-1, peroxiredoxin-2, and thioredoxin (Tables S3 and S4). Concurrently, non-PASC7 was enriched with carbohydrate metabolism (increase levels of α-enolase and UBA52) and vesicle-mediated transport (increased levels of immunoglobulin J chain and UBA52) (Tables S3 and S4).

Altogether, several non-PASC individuals exhibited a profile consistent with active immune and metabolic resolution, characterized by pathways associated with dampened immune signaling and balanced ROS detoxification. Conversely, other non-PASC individuals showed lingering markers of unresolved cellular stress, including enriched apoptotic and senescence pathways linked to elevated linker histones. Collectively, these data indicate that even in young, healthy, non-PASC individuals, the resolution of SARS-CoV-2 infection is not a uniform return to baseline.

## 4. Discussion

The present study aimed to analyze the PBMC-EV proteome to understand the long-term biological impact of long COVID. The PBMC samples reported herein were collected from unvaccinated, young US Marines with a history (lasting >30 days post-acute infection) of PASC headache and drowsiness (one individual), pulmonary PASC symptoms (five individuals), loss of smell (one individual) and without any PASC symptoms (nine individuals) enrolled in CHARM 1.0 and 2.0 clinical studies [6,16]. Our cohorts were mainly comprised of males between 18 and 23 years of age at the time of their first SARS-CoV-2 infection. Therefore, we could not analyze any gender-based differences. Overall, our data suggest that PBMC-EV proteome carry distinctive signatures of interlinked, overlapping molecular cascades imprinted by different PASC symptoms in these young, unvaccinated individuals.

While we acknowledge the limitation of a single-subject analysis or a small sample size, the exceptional degree of PPI enrichment and the biological coherence suggest that our findings are not random. Rather, they seem to represent a highly coordinated physiological state, which should be further validated. This highlights a primary strength of the PBMC-EV approach: its ability to reveal “molecularly rich” signatures even at the individual level, providing a platform for precision medicine where patient stratification is important. In addition, we emphasize that the nano-LC-MS/MS protein hits and enriched biological pathways were not further validated by a secondary method in this study.

Recent clinical oncology studies have highlighted how specific EV size metrics such as D10, D50, and D90 values (representing the size thresholds below which 10%, 50%, and 90% of the EV population falls) can predict clinical treatment outcome [32,33]. Our data also suggest that a lower D50 median PBMC-EV particle size at the baseline (or pre-SARS-CoV-2 infection) may serve as a susceptibility marker of the lingering effects of long COVID. EV particle sizes are determined by factors such as biogenesis pathways, cargo content and cargo diffusion rates [34,35]. EV uptake from recipient cells and their membrane protein composition also vary substantially with size [34,35,36]. These highly heterogeneous EV subpopulations are unlikely to exhibit similar functions due to stoichiometric differences in their volume, surface area and composition [34,35]. For example, even a 50 nm vesicle has 3-fold the surface area and 5-fold the volume of a 30 nm vesicle [34] potentially distributing a higher cargo content or larger size molecules. Thus, the pre-existing size disparities found in PBMC-EVs may reflect baseline alterations in the biological landscape. This, in turn, may correlate with susceptibility to the long-lasting effects of COVID-19 infection.

Many EV-based studies rely on classical exosome transmembrane tetraspanins (CD9, CD63, CD81) as markers during EV enrichment, detection and characterization [20,21]. However, it is now well-known that tetraspanins are heterogeneously distributed in different EV subpopulations [20,21]. Enrichment of different EV subtypes depends heavily on EV isolation methods and the biological source [20,21]. There are many other EV markers and molecular cargo attributed to various EV biogenesis pathways [21]. The predominant identification of metabolic and cytosolic proteins (e.g., GAPDH, actin 1, α-enolase and redoxins), along with the lack of classical tetraspanins in PBMC-EVs, underscores the potential variables such as EV source, isolation method and analytical bias (e.g., untargeted nano-LC-MS/MS) when analyzing EV markers. Furthermore, as mentioned earlier, due to the scarcity of the enriched PBMC-EV samples, we could not perform target-specific identification of CD9, CD63 and CD81 using Western blots. Notably, we observed a temporal shift in the EV marker profile, characterized by a reduction in the number of EV markers found at 6 to 8 months post-infection (T1) compared to the pre-infection baseline (T0) across both PASC and non-PASC individuals. This generalized decline suggests long-lasting alterations in the EV molecular cargo after SARS-CoV-2 infection. Viruses, including SARS-CoV-2, hijack EVs for viral dissemination, persistence and to drive the pathophysiology of the viral infection [37]. Also, EVs participate in anti-viral defense and immunomodulation during viral infections [37]. The EV molecular alterations induced by these events may persist even after the viral clearance depending on the post-infection microenvironment in the body. Additionally, detection of specific EV cargo molecules may correlate with persistent effects of long COVID. For instance, the absence of TIM, a crucial glycolysis enzyme [22] in most of the PASC individuals before and after the SARS-CoV-2 infection, suggests a correlation between altered glycolytic protein signatures and PASC development. Cellular deficiency in TIM is known to impair adenosine triphosphate (ATP) production during glucose consumption, thereby impacting cell survival leading to accumulation of dihydroxyacetone phosphate (DHAP). DHAP degrades to methylglyoxal, a toxic compound that damages cellular DNA, proteins and lipids [38,39]. While our sample size limits broad generalizations, this observation raises the possibility that unique metabolic signatures within PBMC-EVs could serve as PASC biomarkers. Future studies with larger cohorts are warranted to determine if the presence or absence of TIM in PBMC-EV cargo can reliably predict PASC incidence.

The extensive network of peripheral circulation ensures that PBMCs can interact with virtually all tissues, enabling systemic immune surveillance. This process enriches PBMCs with a distinctive array of EVs sequestered from various cellular sources, making them an ideal substrate for extracting complex cellular messages. Our PBMC-EV proteome data suggest that body is in an altered biological state even 6–8 months after the initial SARS-CoV-2 infection, in the participants who reported PASC symptoms lasted >30 days after the infection. One caveat of the T1 PASC clinical survey was that it did not capture whether those same PASC symptoms were present at the time of the survey. Therefore, in the present study, we were only able to delineate the potential long-term impact of the long COVID, while cautioning against potential intermittent infections.

Recent evidence reinforces that PASC is deeply intertwined with diminished antioxidant defenses, oxidative DNA damage, and genomic instability [40,41,42,43]. Furthermore, individuals with PASC exhibit distinctive signatures of brain damage and neurodegeneration linked to AD pathology [44,45]. The individual who reported having PASC-headache and drowsiness (PASC1) had enriched Reactome pathways linked to oxidative stress and DNA damage. Increased accumulation of prelamin A/C in PBMC-EVs of PASC1 was mapped to CDK5 deregulation (Figure 5). Oxidative stress can downregulate the enzymes responsible for processing prelamin A/C [46], impairing the early DNA damage response [47,48,49]. Ultimately, unresolved DNA damage may manifest as defective apoptosis of postmitotic neurons, leading to AD pathology [50,51,52]. This is driven by deregulation and mislocalization of CDK5, a key regulator of cell survival and apoptosis [50,51,52]. DNA damage activates calpain [53], which induces cleavage of p35 into p25, an aberrant activator of CDK5 [54]. Elevated levels of p25 are strongly associated with CDK5 deregulation following exposure to DNA-damaging agents [51].

PASC1 also reported drowsiness and fatigue, with decreased levels of redoxins (peroxiredoxin-2 and thioredoxin) and key glycolytic enzymes, α-enolase, and TIM, mapped to T53-mediated metabolic regulation and glycolysis/gluconeogenesis pathways (Figure 5). CDK5 deregulation can promote oxidative stress and mitochondrial dysfunction by inactivating peroxiredoxin phosphorylation, thereby impairing the ROS detoxification process [55]. Furthermore, previous reports suggest that thioredoxin can modify the redox-sensitive p53 protein [56], which is known to act as a key transcriptional regulator of glycolysis and gluconeogenesis [57], impacting overall cellular energy production pathways. Elevated oxidative stress has been shown to be associated with excessive host energy consumption within PBMC lymphocytes of PASC donors [58]. Impaired fatty acid oxidation and mitochondrial dysfunction seem to play a crucial role in the debilitating fatigue and other sequelae shared between PASC and myalgic encephalomyelitis/chronic fatigue syndrome (ME/CFS) [58,59].

In vascular aging, prelamin A accumulation acts as a primary disruptor of mitosis and a catalyst for DNA damage leading to mitotic failure, genomic instability, and premature senescence [46,47,48]. H1 linker histones stabilize the nucleosome in a higher-order chromatin structure, regulating chromatin accessibility during DNA transcription, replication, and repair [60]. Upon DNA damage, post-translational modifications allow histone H1 to be released from the nucleosome, decondensing chromatin and accelerating the recruitment of DNA damage response factors into the DNA damage sites [60]. When this process fails, cells initiate mechanisms to protect against oncogenic activation by triggering programmed cell death (intrinsic apoptosis) or irreversible cell cycle arrest (cellular senescence) [61]. Loss of H1 linker histones seems to be a characteristic of cellular senescence [62], which aligns with excessive levels of free H1 histones found in our PASC1 PBMC-EVs (Figure 5). Also, extracellular H1 histones may drive neuronal death and neurodegeneration [63]. Senescent cells are metabolically active and develop SASP, releasing a vast array of pro-inflammatory cytokines, chemokines, and growth factors [64]. Endothelial senescence induced by viral infections, including SARS-CoV-2, has been proposed as a potential mechanism for immune dysfunction both in PASC and ME/CFS [65,66]. Markers of cellular senescence and SASP factors have shown to be increased in acute SARS-CoV-2 and PASC [65,66]. SASP provides a chronic source of low-grade inflammation, creating a favorable environment for inappropriate neutrophil activation and degranulation in the absence of an acute pathogenic insult [67]. In addition, elevated S100-A9 levels and lysozyme (LYZ) gene upregulation have been observed in PASC patients [68,69]. Both S100-A9 and lysozyme are associated with many inflammatory conditions, including neuroinflammation and amyloid formation [70,71,72,73].

While SARS-CoV-2 viral persistence may cause DNA damage and genomic instability in PASC [74], we did not detect any viral proteins within our pulmonary PASC cohort. However, the association between phosphorylated SARS-CoV-2 nucleocapsid protein and 14-3-3 isomers in the nucleocytoplasmic shuttling of the N protein has been previously documented [75]. Mechanistically, BAD is one of the BH3 pro-apoptotic proteins, which dephosphorylates and dissociates from 14-3-3 proteins to localize to mitochondria during apoptosis [76]. Conversely, under survival signals, phosphorylated BAD is bound to 14-3-3 scaffold proteins and sequestered in the cytoplasm [77]. During cell cycle arrest, Chk1 phosphorylates Cdc25 isomers, resulting in the direct inactivation of Cdk1, or by promoting Chk1 binding to 14-3-3 proteins to inhibit Cdk1 [78]. Additionally, protein kinase N (PKN), a downstream target of Rho GTPase, can directly inhibit Cdc25c activity by phosphorylation [78]. The accumulation of H3.3 in PBMC-EVs suggests secretion of SASP components associated with cellular senescence [79].

The receptor–ligand interaction between integrin β-3 on platelets and fibrinogen has been shown to activate MAPK signaling through the FAK/Src-Shc-Grb2-SOS signaling axis [80,81]. The amplitude and frequency of cytosolic Ca^2+^ influx dictate the degree of integrin β-3 activation and fibrinogen binding [82]. Specifically, the association of calmodulin-like protein 3, one of the Ca^2+^ sensors, with platelet surface glycoprotein GPIb-IX-V, triggers the Ca^2+^-dependent activation of integrin β-3 [83]. The delayed phase of this integrin-fibrinogen-mediated MAPK activation is important for platelet-mediated clot retraction (clot stabilization) [84]. Accordingly, the decreased T1 abundance of fibrinogen chains, integrin β-3, and calmodulin-like protein 3 observed in pulmonary PASC PBMC-EVs indicates an impairment of platelet activation and clot retraction (Figure 7). Furthermore, previous data suggest that platelet migration relies on a fully functional integrin β-3/Gα13/c-Src/14-3-3 ζ signaling axis to orchestrate Arp2-/3-driven actin nucleation (filament assembly) and lamellipodium formation [85]. This process seems to involve some of the proteins found in PBMC-EVs with decreased T1 abundance, such as actin, vasodilator-stimulated phosphoprotein (e.g., lamellipodium formation) [86] and thymosin-β (e.g., prevent premature actin filament assembly) [87].

Hypercoagulability is widely recognized as a key pathological feature in severe, acute SARS-CoV-2 infection [88]. Nevertheless, some of the acute COVID-19 and PASC patients have shown defects in integrin β-3-mediated platelet activation [89,90]. Mechanistically, the SARS-CoV-2 spike protein can reduce platelet aggregation in whole blood and diminish the activation of integrin β-3 on platelets in presence of spike-neutralizing antibodies [91]. While antibody levels against the spike protein remain a critical variable in PASC patients, impaired integrin β-3 signaling mediated by viral proteins may not be the exclusive driver of platelet dysregulation in these cases. Furthermore, reduced levels of fibrinogen in PBMC-EVs suggest potential sequestration of free fibrin from circulation, contributing to fibrin microclots, which have been previously described in PASC patients [92].

Both fibrinogen and S100-A9 could act as DAMPs in activating TLRs [71,93]. The decreased abundance of S100-A9 within the PBMC-EV proteome suggests a physiological effort to regulate endogenous DAMP molecules. S100-A9 regulates the ROS environment in the ER-phagosome during antigen cross-presentation [94]. As previously noted, S100-A8/A9 act as danger signals during infection-associated inflammation, yet they remain essential for tissue healing and repair [95]. The high levels of S100-A9 observed in some PASC patients may trigger inflammatory cascades via TLR4 or RAGE receptor stimulation [71]. This stimulation leads to the hyperexpression of pro-inflammatory cytokines IL-1β and IL-6 [71] and immune dysregulation reported in PASC patients [2]. Ultimately, the immune system may become desensitized to persistent inflammatory triggers, as evident by the decreased abundance of S100-A9 in some participants who reported pulmonary PASC. While such desensitization might alleviate inflammation and self-perpetuating senescence [96], the resulting decrease in S100-A9 may simultaneously impair the healing and repair of damaged lung endothelial tissues. Beyond its role as a pro-inflammatory alarmin, S100-A9 is a critical mediator of pulmonary tissue repair. It has been shown to directly promote the activation, migration, and extracellular matrix (ECM) production of human lung fibroblasts through the RAGE/MAPK signaling pathway [95].

Pathway analysis of the nine non-PASC individuals provided comparative proteomic signatures to distinguish active resolution from the chronic dysregulation we observed in PASC participants. Absence of PASC symptoms in the non-PASC individuals did not necessarily signify a uniform return to baseline homeostasis months after SARS-CoV-2 infection. Overall, the PBMC-EV proteome data suggest that molecular changes imprinted by PASC are not merely random fluctuations that occurred during the time leading up to the T1 survey. If the changes we observed were random or due to other infections, we would have observed similar trends in non-PASC individuals. With more extensive clinical studies powered by large sample sizes, PBMC-EVs can certainly generate mechanistic biological frameworks, as well as therapeutic or diagnostic biomarkers for complex diseases such as long COVID. Given that our cohort consists of young, active-duty Marines, these findings have direct implications for force health protection and mission readiness. With future studies pursuing PBMC-EV signatures as diagnostic and predictive biomarkers, medical commands can proactively identify biologically vulnerable individuals, tailor specific therapeutic and rehabilitation strategies, and ultimately expedite the safe return to duty of service members, thereby preserving operational readiness.

### Study Limitations

Despite the depth of the proteomic analysis, several limitations must be considered when interpreting these findings. First, the small sample size and narrow age range limit the generalizability of the results and preclude any broad mechanistic conclusions. Because the EV proteome is highly heterogeneous, cohort-level analysis for pulmonary PASC was performed by pooling protein abundance data from five individuals. While pooling data creates a composite molecular network rather than a single-patient profile, this approach was intentionally employed to capture the broader mechanistic landscape imprinted by pulmonary PASC. Given the inherent heterogeneity of long COVID, observing distinct pathway nodes emerging across different individuals allows us to piece together the overarching biological “story”, such as the interplay between platelet dysfunction and immune exhaustion driving long-term consequences of pulmonary sequelae. To maintain full transparency regarding this individual variability, patient-specific fold-changes are detailed in the heatmaps and Supplementary Tables.

While this cohort of young, unvaccinated US Marines provides a unique and controlled look at PASC within a specific demographic, it does not account for the biological variables inherent in genders, vaccinated individuals, older populations, or those with pre-existing comorbidities. Furthermore, viral persistence could not be determined in the present study. This is likely because nano-LC-MS/MS analysis may obscure low-abundance viral proteins.

A common challenge in evaluating ex vivo PBMC-EV generation is the potential for culture-induced stress artifacts, particularly when assessing cells with an inflammatory history. However, our study design effectively controls this through the inclusion of the matched non-PASC cohort. If the distinct proteomic signatures observed in the PASC sub-groups (e.g., oxidative stress, platelet dysfunction) were merely artifacts of post-infection PBMCs reacting differently to cryopreservation or ex vivo culture conditions, these same signatures would be expected to appear uniformly across the non-PASC T1 samples. The absence of these uniform, board-spanning changes in the non-PASC cohort demonstrates that the pathway dysregulations identified are not generic culture artifacts, but rather represent bona fide, phenotype-specific cellular “memory” restricted to individuals experiencing long COVID.

Additionally, the reliance on a polymer-based precipitation method (ExoQuick-TC) for EV enrichment warrants discussion. While polymer-based isolation can co-precipitate non-EV proteins or protein aggregates, it is well-established that all EV isolation techniques involve a trade-off between purity and yield. We specifically chose polymer precipitation to maximize EV yield and reproducibility, which was strictly necessary to generate the comprehensive downstream nano-LC-MS/MS profiles required to build this proof-of-concept mechanistic network. Crucially, we isolated EVs from ex vivo PBMC culture supernatants utilizing exosome-depleted media, rather than directly from plasma. This approach significantly bypasses the heavy lipoprotein and soluble protein contamination typically associated with polymer precipitation of whole blood components, resulting in a cleaner EV fraction. Also, enriched EVs were further passed through resin-based EV purification columns to increase the purity. Nonetheless, future studies expanding upon this biological framework should look to incorporate targeted secondary validations (e.g., Western blot or ELISA) to confirm key mechanistic nodes.

Finally, although the temporal shift from T0 (pre-infection) to T1 (6–8 months post-infection) indicates long-lasting alterations, further studies are required to determine direct association with PASC using immediate time points.

## Data Availability

The nano-LC/MS/MS raw data have been deposited to the ProteomeXchange Consortium via the PRIDE partner repository with the dataset identifier PXD081034 and 10.6019/PXD081034. The original contributions presented in this study are included in the article/Supplementary Material. Further inquiries can be directed to the corresponding author.

## Supplementary Materials

The following supporting information can be downloaded at https://www.mdpi.com/article/10.3390/cells15211969/s1, Figure S1: Significantly enriched pathways associated with increased T1 abundance in PASC individuals; Figure S2: Significantly enriched pathways associated with decreased T1 abundance in PASC individuals. Table S1: Patient demographics; Table S2: Viability of freeze-thawed PBMCs before culturing and after harvesting EVs; Table S3: Fold-change of abundance of the proteins at T1 in PASC and non-PASC individuals; Table S4: Enriched Reactome pathways mapped to proteins showing increased and decreased abundance in non-PASC individuals.

## Abbreviations

The following abbreviations are used in this manuscript:

AD	Alzheimer’s Disease
BAD	Bcl-2-Associated Death Promoter
BH3	Bcl-2 Homology 3
CDK5	Cyclin-Dependent Kinase 5
CHARM	COVID-19 Health Action Response for Marines
Chk1/Chk2	Checkpoint Kinase 1/2
DAMPs	Danger-Associated Molecular Patterns
ELISA	Enzyme-Linked Immunosorbent Assay
ER	Endoplasmic Reticulum
EVs	Extracellular Vesicles
FDR	False Discovery Rate
GAPDH	Glyceraldehyde 3-Phosphate Dehydrogenase
MAPK	Mitogen-Activated Protein Kinase
ME/CFS	Myalgic Encephalomyelitis/Chronic Fatigue Syndrome
Nano-LC/MS/MS	Nanospray Liquid Chromatography Coupled with Tandem Mass Spectrometry
NMRC	Naval Medical Research Command
NTA	Nanoparticle Tracking Analysis
OD	Optical Density
PASC	Post-Acute Sequelae of SARS-CoV-2
PBMC-EVs	EVs Released from PBMCs
PBMCs	Peripheral Blood Mononuclear Cells
PBS	Phosphate-Buffered Saline
PKN	Protein Kinase N
PLK	Polo-Like Kinase 1
PPI	Protein–Protein Interaction
PSM	Peptide Spectrum Match
ROS	Reactive Oxygen Species
SAHF	Senescence-Associated Heterochromatin Foci
SASP	Senescence-Associated Secretory Phenotype
TIM	Triosephosphate Isomerase
TLR	Toll-Like Receptor
UBA52	Ubiquitin Ribosomal Protein eL40 Fusion Protein
US	United States
WRAIR	Walter Reed Army Institute of Research
