# Supplementary material for: Extracellular Vesicle Protein Cargo Reveals Potential Long-Term Biological Impact of Long COVID
Source: Cells. 2026 Oct 29;15(21):1969. doi: 10.3390/cells15211969 (PMC13761425; doi:10.3390/cells15211969)
Supplement: Supplementary file 1 [file cells-15-01969-s001.zip › Table S1.pdf]

Table S1: Participant demographics

				First SARS-CoV-2 infection			PASC		
**Participant code**	**Age**	**Gender**	**Current smoker**	**Viral load on the first day of PCR + (ORF1ab CT value)**	**Symptomatic /Asymptomatic**	**Symptoms reported during the first infection**	**Duration between T0 (pre-infection) and T1 (PASC survey 6-8 months post-infection)**	**PASC symptoms lasted more than 30 days after initial SARS-CoV-2 infection**	**Vaccination status at the time of T1 survey**
PASC 1	18	M	No	19.9	Symptomatic	Muscle aches, Fatigue, Headache, Runny nose, Sore throat	225	Drowsy (Fatigue), Headache	Not Vaccinated
PASC 2	18	M	No	26.07	Asymptomatic	N/A	217	Congestion (Nose-Throat-Chest)	Not Vaccinated
PASC 3	18	M	No	22	Symptomatic	Muscle aches, Fatigue, Runny nose, Cough, Shortness of breath	259	Shortness of breath	Not Vaccinated
PASC 4	18	M	No	26.21	Symptomatic	Subjective fever, Chills, Muscle aches, Fatigue, Runny nose, Sore throat, Shortness of breath, Headache	205	Shortness of breath	Not Vaccinated
PASC 5	20	M	Yes	35.76	Symptomatic	Runny nose	196	Slower running times, Nose-Throat-Chest	Not Vaccinated
PASC 6	18	F	No	19.49	Symptomatic	Subjective fever, Chills, Muscle aches, Fatigue, Nausea or vomiting, Decreased ability to taste or smell, Abdominal pain, Headache	182	Shortness of breath	Not Vaccinated
PASC 7	19	M	No	28.14	Symptomatic	Decreased ability to taste or smell	245	Loss of smell	Not Vaccinated
PASC 8	18	M	Yes	19.91	Asymptomatic	N/A	184	Loss of smell and taste, flu like symptoms, Nose-Throat-Chest	Not Vaccinated
Non-PASC 1	18	M	No	20.82	Asymptomatic	N/A	215	None	Not Vaccinated
Non-PASC 2	18	M	Yes	35.1	Asymptomatic	N/A	196	None	Not Vaccinated
Non-PASC 3	23	M	No	17.72	Symptomatic	Muscle aches, Sore throat, Cough, Shortness of breath, Headache	246	None	Completed the second vaccination dose on the day of the T2 survey
Non-PASC 4	19	M	No	30.75	Asymptomatic	N/A	182	None	Not Vaccinated
Non-PASC 5	19	F	Yes	>37	Symptomatic	Subjective fever, Muscle aches, Fatigue, Headache	182	None	Not Vaccinated
Non-PASC 6	19	F	No	26.24	Asymptomatic	N/A	183	None	Not Vaccinated
Non-PASC 7	18	F	No	28.16	Asymptomatic	N/A	185	None	Not Vaccinated
Non-PASC 8	21	F	No	16.74	Symptomatic	Subjective fever, Chills, Runny nose, Headache	183	None	Not Vaccinated
Non-PASC 9	18	F	No	18.8	Symptomatic	Subjective fever, Muscle aches, Fatigue, Runny nose, Sore throat, Headache, Shortness of Breath, Loss of Taste	181	None	Not Vaccinated

N/A - Not Applicable
M - Male, F - Female
PASC - Individuals with a history of PASC symptoms lasted more than 30 days after initial SARS-CoV-2 infection
Non-PASC - Individuals with no history of PASC symptoms after the initial SARS-CoV-2 infection
CT values represent the viral load on the first day of viral detection by PCR during the first SARS-CoV-2 infection
T0 - Pre-infection
T1 - The time point of PASC Survey and Sample collection
