# Supplementary material for: Extracellular Vesicle Protein Cargo Reveals Potential Long-Term Biological Impact of Long COVID
Source: Cells. 2026 Oct 29;15(21):1969. doi: 10.3390/cells15211969 (PMC13761425; doi:10.3390/cells15211969)
Supplement: Supplementary file 1 [file cells-15-01969-s001.zip › Table S2.pdf]

**Table S2: Viability of freeze-thawed PBMCs before culturing and after harvesting EVs**

| Participant code | Time point | PBMC viability (%) Before Plating | PBMC viability (%) After EV harvesting | Total number of cells plated | Total number of cells harvested | EV concentration (particles/mL) |
|---|---|---|---|---|---|---|
| PASC 1 | T0 | 94.9% | 81.4% | 1.36E+07 | 8.00E+06 | 3.44E+08 |
| | T1 | 97.0% | 85.7% | 2.07E+07 | 1.84E+07 | 1.20E+09 |
| PASC 2 | T0 | 97.4% | 78.7% | 1.39E+07 | 1.07E+07 | 5.56E+08 |
| | T1 | 94.2% | 89.4% | 1.10E+07 | 8.02E+06 | 5.18E+08 |
| PASC 3 | T0 | 96.7% | 79.8% | 7.18E+06 | 6.84E+06 | 6.40E+08 |
| | T1 | 95.8% | 80.8% | 7.28E+06 | 6.24E+06 | 4.88E+08 |
| PASC 4 | T0 | 95.5% | 77.4% | 1.30E+07 | 7.00E+06 | 6.68E+08 |
| | T1 | 96.1% | 85.3% | 8.59E+06 | 4.40E+06 | 5.40E+08 |
| PASC 5 | T0 | 94.6% | 82.3% | 7.05E+06 | 5.70E+06 | N/A |
| | T1 | 95.6% | 90.2% | 1.16E+07 | 8.86E+06 | 9.48E+08 |
| PASC 6 | T0 | 90.8% | 37.9% | 4.30E+06 | 6.50E+05 | N/A |
| | T1 | 93.7% | 84.5% | 1.04E+07 | 6.14E+06 | 5.90E+08 |
| PASC 7 | T0 | 96.3% | 66.6% | 7.12E+06 | 2.78E+06 | 8.82E+08 |
| | T1 | 91.6% | 78.7% | 2.23E+06 | 6.30E+05 | 1.21E+09 |
| PASC 8 | T0 | 93.6% | 78.4% | 1.51E+07 | 2.98E+06 | 2.38E+08 |
| | T1 | 94.9% | 82.1% | 3.25E+06 | 6.34E+06 | 5.42E+08 |
| Non-PASC 1 | T0 | 80.2% | 54.5% | 3.20E+06 | 5.86E+05 | 5.70E+08 |
| | T1 | 94.5% | 93.3% | 4.74E+06 | 3.66E+06 | 6.14E+08 |
| Non-PASC 2 | T0 | 96.2% | 86.5% | 8.84E+06 | 3.50E+06 | N/A |
| | T1 | 96.2% | 94.3% | 1.47E+07 | 6.48E+06 | 8.14E+08 |
| Non-PASC 3 | T0 | 97.4% | 84.5% | 8.20E+06 | 3.62E+06 | N/A |
| | T1 | 94.5% | 92.7% | 1.25E+06 | 4.98E+05 | N/A |
| Non-PASC 4 | T0 | 91.6% | 90.5% | 5.90E+06 | 5.00E+06 | N/A |
| | T1 | 95.0% | 92.0% | 8.76E+06 | 6.20E+06 | 5.00E+08 |
| Non-PASC 5 | T0 | 90.6% | 84.4% | 7.51E+06 | 6.46E+06 | 9.64E+08 |
| | T1 | 77.6% | 65.8% | 1.02E+07 | 8.02E+06 | 1.46E+09 |
| Non-PASC 6 | T0 | 93.1% | 84.8% | 7.52E+06 | 8.40E+06 | 1.14E+09 |
| | T1 | 90.6% | 84.1% | 1.57E+07 | 1.34E+07 | 1.28E+09 |
| Non-PASC 7 | T0 | 95.6% | 90.6% | 7.36E+06 | 6.14E+06 | 7.98E+08 |
| | T1 | 94.4% | 91.6% | 8.31E+06 | 7.42E+06 | 5.88E+08 |
| Non-PASC 8 | T0 | 94.3% | 80.9% | 7.84E+06 | 6.70E+06 | 3.30E+08 |
| | T1 | 94.1% | 85.5% | 1.21E+07 | 1.22E+07 | 7.66E+08 |
| Non-PASC 9 | T0 | 93.0% | 83.5% | 5.91E+06 | 8.10E+06 | 1.09E+09 |
| | T1 | 90.3% | 88.0% | 1.17E+07 | 1.12E+07 | 4.98E+08 |

T0 - Pre-infection (before initial SARS-CoV-2 infection)
T1 - Time point of PASC survey and sample collection (6-8 months post-infection)
PASC - Individuals with a history of PASC symptoms lasted more than 30 days after initial SARS-CoV-2 infection
Non-PASC - Individuals with no history of PASC symptoms after the initial SARS-CoV-2 infection
NA - Not available
Before plating : The viability was taken immeditely after freeze-thawing the PBMCs, and before plating them on 96 well plates
Before harevsting: The cell viability was taken after harvesting supernatunt for EV enrichment (following 22h of incubation at 37°C/5% CO2)

**Descriptive statistics of PBMC viability, counts, and EV particle concentration**

| | Before Plating (%) | After EV harvesting (%) | Total number of cells plated | Total number of cells harvested | EV concentration (particles/mL) |
|---|---|---|---|---|---|
| | | | | | |
| **Minimum** | 77.6 | 37.9 | 1.25E+06 | 4.98E+05 | 2.38E+08 |
| **Maximum** | 97.4 | 94.3 | 2.07E+07 | 1.84E+07 | 1.46E+09 |
| **Range** | 19.8 | 56.4 | 1.95E+07 | 1.79E+07 | 1.22E+09 |
| | | | | | |
| **Mean** | 93.47 | 81.96 | 9.00E+06 | 6.51E+06 | 7.42E+08 |
| **Std. Deviation** | 4.205 | 11.34 | 4.26E+06 | 3.82E+06 | 3.15E+08 |
| **Std. Error of Mean** | 0.7212 | 1.945 | 7.31E+05 | 6.54E+05 | 5.95E+07 |
