# Supplementary material for: Extracellular Vesicle Protein Cargo Reveals Potential Long-Term Biological Impact of Long COVID
Source: Cells. 2026 Oct 29;15(21):1969. doi: 10.3390/cells15211969 (PMC13761425; doi:10.3390/cells15211969)
Supplement: Supplementary file 1 [file cells-15-01969-s001.zip › Table S3.pdf]

Table S3: Fold-change of abundance of the proteins at T1 in PASC and non-PASC individuals

			Headache & drowsiness	Pulmonary PASC					Loss of smell	Non-PASC								
**Entry Name**	**Protein names**	**Protein ID**	**PASC1**	**PASC2**	**PASC3**	**PASC4**	**PASC5**	**PASC6**	**PASC7**	**Non-PASC1**	**Non-PASC2**	**Non-PASC3**	**Non-PASC4**	**Non-PASC5**	**Non-PASC6**	**Non-PASC7**	**Non-PASC8**	**Non-PASC9**
1433S_HUMAN	14-3-3 protein sigma	P31947				4.190												
1433Z_HUMAN	14-3-3 protein zeta/delta	P63104		1.076		0.391												
ACTB_HUMAN	Actin, cytoplasmic 1	P60709	11.314	0.475		0.603		0.971			2.127	0.037		0.257	1.471			
ACTH_HUMAN	Actin, gamma-enteric smooth muscle	P63267															4.051	
SAHH_HUMAN	Adenosylhomocysteinase (AdoHcyase)	P23526				1.193												
FETUA_HUMAN	Alpha-2-HS-glycoprotein	P02765	15.117	0.409	1.643	0.335	0.091	1.304	0.788	13.076	1.180	0.035	1.020	0.202	8.724	1.737	5.894	14.917
ENOA_HUMAN	Alpha-enolase	P06733	0.588			0.409						0.023				2.185	0.478	
FETA_HUMAN	Alpha-fetoprotein	P02771	0.041															
ANXA1_HUMAN	Annexin A1	P04083	13.067															
ANXA2_HUMAN	Annexin A2	P07355													0.544	0.428		
ARGI1_HUMAN	Arginase-1	P05089	3.645							0.733		0.030			0.250	0.366	0.313	
CHD5_HUMAN	ATP-dependent chromatin remodeler CHD5	Q8TDI0			0.175	1.815												
E41LA_HUMAN	Band 4.1-like protein 4A	Q9HCS5					2.320		0.327									
BLMH_HUMAN	Bleomycin hydrolase (BH)	Q13867				0.078				2.303		2.046						
BASP1_HUMAN	Brain acid soluble protein 1	P80723					0.248	1.052						1.390				
CALL3_HUMAN	Calmodulin-like protein 3	P27482				0.455												
CATA_HUMAN	Catalase	P04040	0.293							1.900	0.599	0.467	2.148	0.204	0.400	0.460		
CATD_HUMAN	Cathepsin D	P07339	1.032			0.007				3.319		0.218	1.297		0.318	0.741		
CAVN2_HUMAN	Caveolae-associated protein 2	O95810				0.522									0.442			
CDKL4_HUMAN	Cyclin-dependent kinase-like 4	Q5MAI5						0.179										
CYTA_HUMAN	Cystatin-A	P01040									0.015	2.365	0.433		0.502	0.547	6.958	
CYTM_HUMAN	Cystatin-M	Q15828				0.585								0.802		1.310		
CRCT1_HUMAN	Cysteine-rich C-terminal protein 1	Q9UGL9				0.271												
EF1A2_HUMAN	Elongation factor 1-alpha 2	Q05639	0.287															
BIP_HUMAN	Endoplasmic reticulum chaperone BiP	P11021										0.237						
ECM1_HUMAN	Extracellular matrix protein 1	Q16610	0.833							2.981						0.553		
FBX50_HUMAN	F-box only protein 50	Q6ZVX7	0.995									0.251			0.480	0.260	0.691	
FIBA_HUMAN	Fibrinogen alpha chain	P02671		0.468														
FIBB_HUMAN	Fibrinogen beta chain	P02675		1.207														
FIBG_HUMAN	Fibrinogen gamma chain	P02679		0.171														
LEG7_HUMAN	Galectin-7	P47929				0.270										0.900		
GGCT_HUMAN	Gamma-glutamylcyclotransferase	O75223	0.431			0.020		0.464	0.763	6.056	0.466	0.482		0.351	0.396	0.744	0.746	
GSDMA_HUMAN	Gasdermin-A (Gasdermin-1)	Q96QA5		1.490														
G3P_HUMAN	Glyceraldehyde-3-phosphate dehydrogenase (GAPDH)	P04406	3.290			0.031		1.479			0.479	0.825	2.818		0.557	0.519	0.858	
HSPB1_HUMAN	Heat shock protein beta-1	P04792				0.344												
HSP72_HUMAN	Heat shock-related 70 kDa protein 2	P54652											1.842					
HBA_HUMAN	Hemoglobin subunit alpha	P69905															0.575	
HBB_HUMAN	Hemoglobin subunit beta	P68871							2.722	0.975	1.498		0.386					
H12_HUMAN	Histone H1.2	P16403	66.703	0.367		2.233								1.654	1.983			
H14_HUMAN	Histone H1.4	P10412		0.422		1.326			0.344						2.538			
H15_HUMAN	Histone H1.5	P16401	18.475											1.874	4.409			
H2B2E_HUMAN	Histone H2B type 2-E	Q16778	6.338															
H33_HUMAN	Histone H3.3	P84243				8.277												
IGHA1_HUMAN	Immunoglobulin heavy constant alpha 1	P01876						0.741										
IGJ_HUMAN	Immunoglobulin J chain	P01591														1.258		
IGKC_HUMAN	Immunoglobulin kappa constant	P01834	2.081	13.145	0.130	0.149								1.224		0.732	0.359	
IGLC2_HUMAN	Immunoglobulin lambda constant 2	P0DOY2									0.209							
ITB3_HUMAN	Integrin beta-3	P05106				0.694												
IDHC_HUMAN	Isocitrate dehydrogenase [NADP]	O75874	0.591															
LYSC_HUMAN	Lysozyme C	P61626	6.820		0.165	0.296	0.691		0.152			0.063	0.370			0.351		
MT1X_HUMAN	Metallothionein-1X	P80297					0.299	2.251			1.238			0.390		0.351		
MUC19_HUMAN	Mucin-19 (MUC-19)	Q7Z5P9									1.863		1.026					
AHNK_HUMAN	Neuroblast differentiation-associated protein AHNAK (Desmoyokin)	Q09666		1.133								2.428						
PRDX1_HUMAN	Peroxiredoxin-1	Q06830	2.082						0.586		0.333	0.038	0.184			0.460		
PRDX2_HUMAN	Peroxiredoxin-2	P32119	0.546									0.497	1.440		0.317	0.309	0.440	
LMNA_HUMAN	Prelamin-A/C	P02545	3.524															
PIP_HUMAN	Prolactin-inducible protein	P12273										0.769						
LRP1_HUMAN	Prolow-density lipoprotein receptor-related protein 1 (LRP-1)	Q07954																1.177
PSA3_HUMAN	Proteasome subunit alpha type-3	P25788										0.557				0.573		
PSA6_HUMAN	Proteasome subunit alpha type-6	P60900									2.761	1.557						
PSB6_HUMAN	Proteasome subunit beta type-6	P28072										0.594						
PACN2_HUMAN	Protein kinase C and casein kinase substrate in neurons protein 2	Q9UNF0								1.510								
POF1B_HUMAN	Protein POF1B	Q8WVV4				4.393					0.549	0.090	1.332		0.241	0.641	0.882	
S10AE_HUMAN	Protein S100-A14	Q9HCY8								0.829						0.711		
S10AG_HUMAN	Protein S100-A16	Q96FQ6	2.485															
S10A6_HUMAN	Protein S100-A6	P06703				0.038												
S10A8_HUMAN	Protein S100-A8	P05109										0.086		0.418				
S10A9_HUMAN	Protein S100-A9	P06702	56.721					0.281				0.276	0.076	0.096		0.177		
PTMA_HUMAN	Prothymosin alpha	P06454						2.335						1.545	7.002			
AXA2L_HUMAN	Putative annexin A2-like protein	A6NMY6		4.615	1.831		0.710		1.146									
RFIP4_HUMAN	Rab11 family-interacting protein 4	Q86YS3								0.562								
TRFE_HUMAN	Serotransferrin (Transferrin)	P02787	0.243															
SPB12_HUMAN	Serpin B12	Q96P63	2.184								0.173	0.133	0.989		0.428	0.581	0.327	
SPR2D_HUMAN	Small proline-rich protein 2D	P22532										0.198					0.688	
SLK_HUMAN	STE20-like serine/threonine-protein kinase (STE20-like kinase)	Q9H2G2					0.593		1.194									
STX3_HUMAN	Syntaxin-3	Q13277																0.697
THIO_HUMAN	Thioredoxin (Trx)	P10599	0.125					0.915	0.887							0.727		
TYB4_HUMAN	Thymosin beta-4	P62328		0.353	0.757	7.500								0.484		0.739		79.817
SPT6H_HUMAN	Transcription elongation factor SPT6 (hSPT6)	Q7KZ85	9.414													0.953		
TAD2B_HUMAN	Transcriptional adapter 2-beta	Q86TJ2										1.182						
TPIS_HUMAN	Triosephosphate isomerase (TIM)	P60174	0.714									0.071	1.043					
TBA1A_HUMAN	Tubulin alpha1A chain	Q71U36	1.790															
TBB5_HUMAN	Tubulin beta chain	P07437	5.219															
TNR1A_HUMAN	Tumor necrosis factor receptor superfamily member 1A	P19438								0.027	0.139	0.722	0.291					
RL40_HUMAN	Ubiquitin-ribosomal protein eL40 fusion protein (UBA52)	P62987	2.680					0.375	1.657	0.486	0.796	0.087	0.864		0.569	1.173	0.566	
VASP_HUMAN	Vasodilator-stimulated phosphoprotein (VASP)	P50552			0.987	0.676	0.748							1.178	1.265	0.959		
VIME_HUMAN	Vimentin	P08670	13.223															
VTDB_HUMAN	Vitamin D-binding protein	P02774											1.852					
ZA2G_HUMAN	Zinc-alpha-2-glycoprotein	P25311	0.942								0.432	0.459	0.874		1.147	0.464	0.687	

Fold-change is only shown for the proteins which were present in both T0 and T1.
